# Discovery and Characterization of Polymyxin-Resistance Genes *pmrE* and *pmrF* from Sediment and Seawater Microbiome

**DOI:** 10.1128/spectrum.02736-22

**Published:** 2023-01-05

**Authors:** Hwanjin Joo, Hyunuk Eom, Youna Cho, Mina Rho, Woon Ju Song

**Affiliations:** a Department of Chemistry, Seoul National University, Seoul, Republic of Korea; b Department of Computer Science, Hanyang University, Seoul, Republic of Korea; c Department of Biomedical Informatics, Hanyang University, Seoul, Republic of Korea; Nanjing Agricultural University

**Keywords:** polymyxin, antibiotic resistance, metagenome, UDP-glucose dehydrogenase, undecaprenyl-phosphate 4-deoxy-4-formamido-l-arabinose transferase

## Abstract

Polymyxins are the last-line antibiotics used to treat Gram-negative pathogens. Thus, the discovery and biochemical characterization of the resistance genes against polymyxins are urgently needed for diagnosis, treatment, and novel antibiotic design. Herein, we report novel polymyxin-resistance genes identified from sediment and seawater microbiome. Despite their low sequence identity against the known *pmrE* and *pmrF*, they show *in vitro* activities in UDP-glucose oxidation and l-Ara4N transfer to undecaprenyl phosphate, respectively, which occur as the part of lipid A modification that leads to polymyxin resistance. The expression of *pmrE* and *pmrF* also showed substantially high MICs in the presence of vanadate ions, indicating that they constitute polymyxin resistomes.

**IMPORTANCE** Polymyxins are one of the last-resort antibiotics. Polymyxin resistance is a severe threat to combat multidrug-resistant pathogens. Thus, up-to-date identification and understanding of the related genes are crucial. Herein, we performed structure-guided sequence and activity analysis of five putative polymyxin-resistant metagenomes. Despite relatively low sequence identity to the previously reported polymyxin-resistance genes, at least four out of five discovered genes show reactivity essential for lipid A modification and polymyxin resistance, constituting antibiotic resistomes.

## INTRODUCTION

Antibiotic resistance poses a global threat to human health. It continuously emerges and rapidly spreads among pathogens, leading to the development of multidrug-resistant bacteria; they were detected nearly everywhere, including the USA, Canada, South America, Europe, Africa, the Middle East, Southeast Asia, and Australia (1). As a result, Centers for Disease Control and Prevention reported that more than 2.8 million people were infected by antibiotic-resistant pathogens and 35 thousand people deceased in the USA (2), necessitating the discovery of novel antibiotics to replace preexisting ones.

However, we have faced the so-called antibiotic resistance paradox (3). If a new antibiotic presented low efficacy, commercial sales would reduce, and with high efficacy, the usage would be restrained to preserve its resistant-free activity. Consequently, the development of novel antibiotics is not profitable for the pharmaceutical industry in both cases, and the number of antibiotics approved by the US Food and Drug Administration has continuously decreased over the past 3 decades (4).

As an alternative, preexisting antibiotics with low levels of resistance can be reassessed. Along this line, polymyxins, such as polymyxin B and E (also known as colistin), can be of interest (5–8). They are composed of cyclic and cationic nonribosomal peptides produced by the Gram-positive bacterium Paenibacillus polymyxa (see Fig. S1A and B in the supplemental material) (9). These secondary metabolites bind to lipopolysaccharides (LPS) on the outer membrane via electrostatic and hydrophobic interactions (10, 11), subsequently disrupting the membranes of Gram-negative bacteria (12–14). They were discovered in the 1940s (15), but their use was abandoned or restricted in clinical practice in the 1960s due to nephrotoxicity (16, 17). Recently, they reemerged as the last-line antibiotics to treat infections of multidrug-resistant or extensively drug-resistant Gram-negative bacteria. In the meantime, however, polymyxins have been used for animal growth promotion and agriculture (18–20), subsequently developing polymyxin-resistant strains (21–25). These circumstances suggest that an up-to-date detection and analysis of polymyxin-resistance genes are required (26, 27).

The proposed molecular mechanism of polymyxin resistance is associated with modifying LPS. Various conditions, such as low pH, low Mg^2+^, and high Fe^3+^/Al^3+^, can function as chemical stimuli (13, 14, 28–30) to two-component systems, PhoP/PhoQ and PmrA/PmrB. They upregulate seven genes, in *pmr* operon (*pmrHFIJKLM*), also called *arn* operon (*arnBCADTEF*), and *pmrE* (Fig. S1C). Then, the gene cluster modifies the phosphate group of lipid A in LPS with a cationic molecule, such as 4-amino-4-deoxy-l-arabinose (l-Ara4N). Consequently, the modified lipid A shows substantially weaker interactions with positively charged polymyxins, conferring the antibiotic resistance. Although they are latent under normal cell-growth conditions, these genes are present in several Gram-negative bacteria, suggesting that the activation and emergence of these chemical processes may occur more often and rapidly than expected.

Integrative studies of bioinformatic and biochemical analyses can be a powerful approach to discover and characterize novel genes related to antibiotic resistance (31–33). Particularly, the discovery and biochemical validation of articulately sorted genes from metagenomes have allowed us to explore different sequence variations apart from genomes. In addition, biochemical characterization enabled us to validate the chemical activity of novel functional genes.

Herein, we carried out integrated bioinformatics and biochemical analyses of two discrete genes related to polymyxin resistance. We discovered three putative *pmrE* genes and two putative *pmrF* genes from various environmental samples, where both *pmrE* and *pmrF* were involved in modifying LPS, severely weakening the antibiotic action of polymyxins. The resulting *pmrE* was also annotated as uridine-diphosphate (UDP)-glucose dehydrogenase or UDP-glucose 6-dehydrogenase (UGDH) because it catalyzed the sequential oxidation of UDP-glucose into UDP-glucuronic acid (34–38) (Fig. S1C). *PmrF* (*arnC* or *yfbF*) was also annotated as undecaprenyl-phosphate 4-deoxy-4-formamido-l-arabinose transferase, which transferred l-Ara4FN group to undecaprenyl phosphate (UndP) (Fig. S1C). Although either *pmrE* or *pmrF* alone may not be biologically active, the discovery and characterization of individual genes at the molecular levels can provide in-depth knowledge of polymyxin resistomes. Thus, the genes were heterologously expressed for biochemical characterization, and their biochemical activities were determined under both *in vitro* and *in vivo* conditions. Our work demonstrated that the discovered genes are chemically competent in modifying lipid A, suggesting potential roles in polymyxin resistance.

## RESULTS AND DISCUSSION

### Novel polymyxin-resistance genes.

Polymyxin-resistant genes were collected from the sediment and seawater microbiome samples. After clustering, we discovered five putative pmr genes (*pmrE2*, *pmrE3*, *pmrE4* and *pmrF2*, *pmrF3*) (see Table S1 in the supplemental material). We also prepared each gene in E. coli (*pmrE1* and *pmrF1*) as a control. The *pmrE2*, *pmrE3*, and *pmrE4* genes showed 64%, 39%, and 41% sequence identities to *pmrE1*, respectively (Table S2). Sequence network analysis showed that the discovered metagenomic *pmrE* genes were considerably dissimilar from the previously reported UGDHs (Fig. S2), suggesting that biochemical studies of these genes may expand the scope of our understanding of UGDH genes. NCBI BLAST sequence analysis indicated that *pmrE2*, *pmrE3*, and *pmrE4* genes are most close to UGDH or nucleotide sugar dehydrogenase from Celeribacter persicus, *Candidatus Methanofastidiosum* sp., and Pseudoxanthomonas suwonensis, respectively (78%, 44%, and 45% sequence identity). Notably, the *pmrE3* gene is highly similar to the marine metagenome samples collected from the Eastern North American coast to the Eastern Pacific Ocean (93% sequence identity) (39).

**FIG 1:**
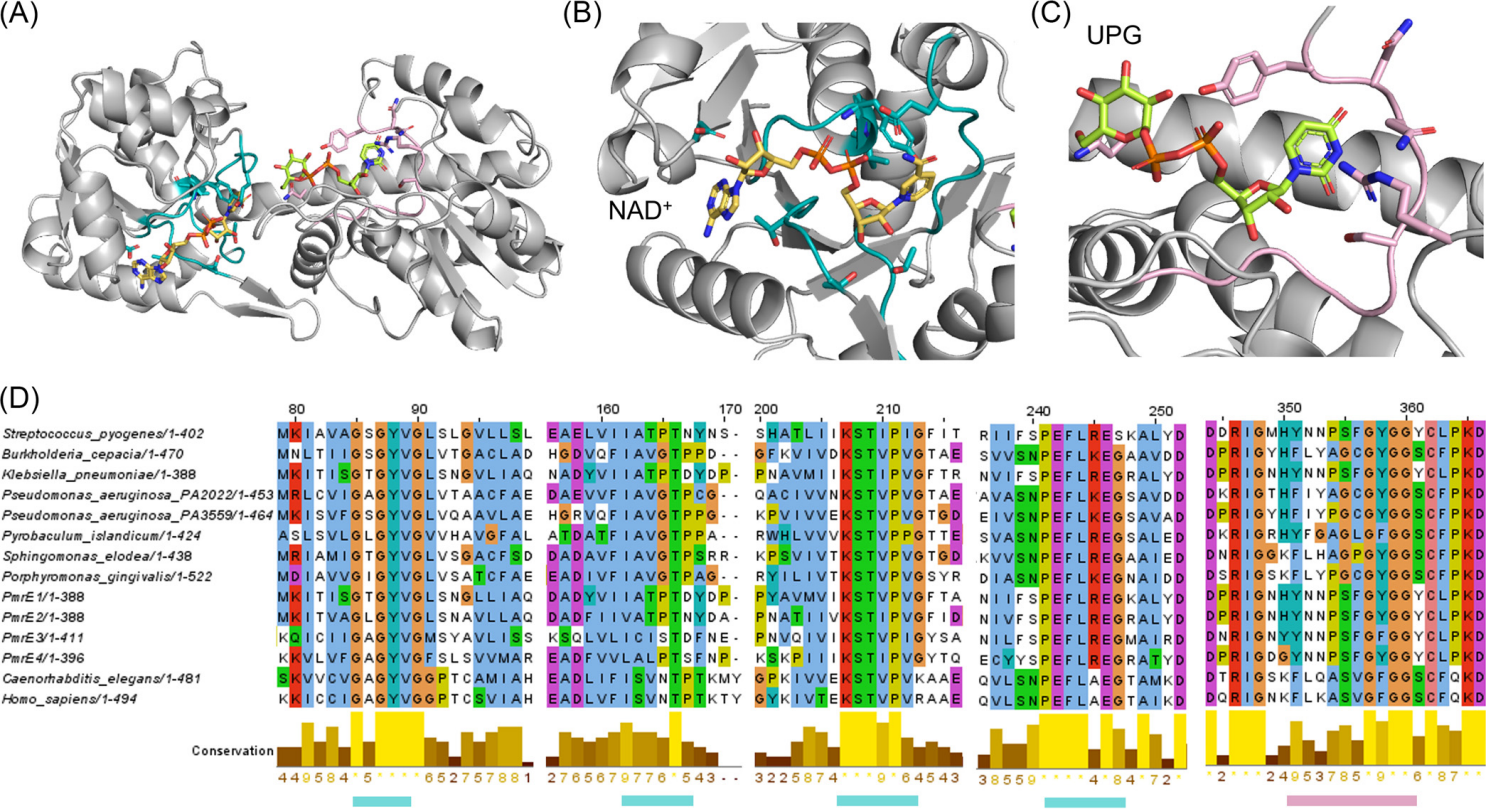
Structure-guided sequence analysis of genes *pmrE1* to *pmrE4*. (A) The substrate-binding pocket of PmrE1. The structure was simulated by SWISS-model using the crystal structure of UGDH from Klebsiella pneumoniae (PDB 3PLR) and UDP-glucose from Homo sapiens (PDB 2Q3E). NAD^+^ and UDP-glucose-binding domains are colored in cyan and light magenta, respectively. The enlarged region of (B) NAD^+^- and (C) UPG-binding domains from panel A are shown. (D) Multiple sequence alignment, representing the NAD^+^-binding domain (cyan bar) and UDP-glucose-binding domain (light magenta bar).

We constructed homology modeling structures of the putative *pmrE* genes and inspected the sequences and structures of the previously reported UGDHs (Fig. 1A to C and Fig. S3). The *pmrE1 to pmrE4* genes showed highly conserved sequence motifs that encoded two active sites to bind substrates, NAD^+^ and UDP-glucose. The NAD^+^-binding site is composed of four sequence motifs: GxGYV, I(A|S)(V|T)(G|P)T(P|D), KST(V|I)P(V|I), and PEFL(R|K|A)EG (Fig. 1B and D). The sequence motif for the UDP-glucose-binding site was also preserved in all *pmrE* genes as (Y|F)xx(P|A)(S|G)xG(Y|F)GG (Fig. 1C to D) (36, 37, 40, 41).

We also explored the sequences and homology modeling structures of the putative *pmrF* genes. Few *pmrF*-like genes have been reported to date; only five have been deposited in the UniProtKB sequence database, and they were from E. coli or Yersinia pseudotuberculosis. In addition, to the best of our knowledge, no biochemical characterization was conducted, not even with *pmrF* from E. coli. Additionally, no protein structure of the same Enzyme Commission (EC) number as PmrF (EC 2.4.2.53) is available in the RCSB database to date. The *pmrF2* and *pmrF3* genes showed 41% and 40% sequence identities to *pmrF1*, respectively (Table S2), and they were similar to glycosyltransferases from *Gammaproteobacteria* and *Chloroflexi* species with a sequence identity of 62% and 72%, respectively. Homology modeling suggests that PmrF is similar to polyisoprenyl-phosphate glycosyltransferase GtrB from *Synechocystis* sp. *PCC6803* (PDB 5EKP), which shows 26 to 32% protein sequence identity to PmrF1 to PmrF3 (42).

**FIG 2:**
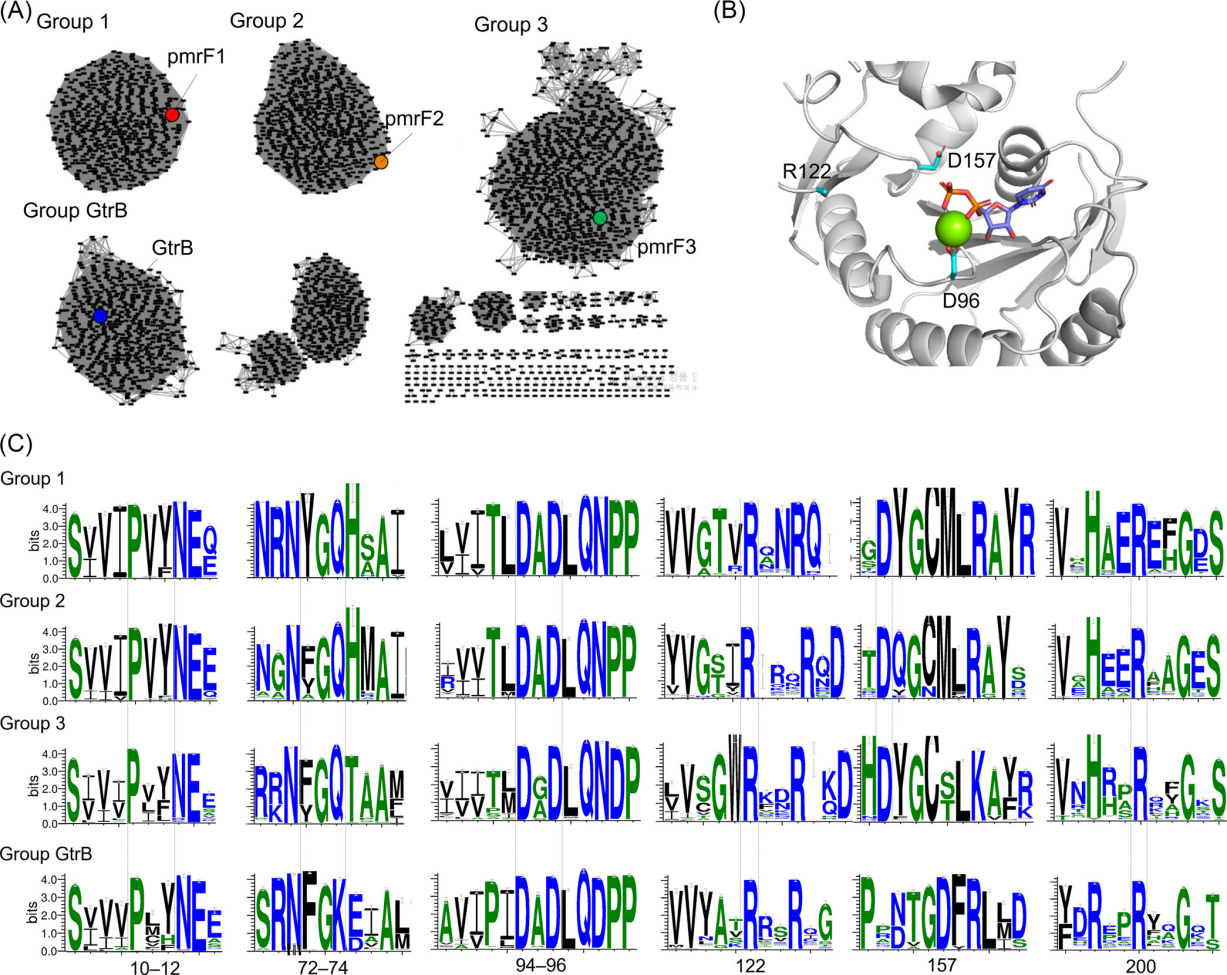
Sequence and structure analysis of PmrF-like proteins. (A) Sequence similarity network analysis of PmrF1 to PmrF3 and GtrB. Each node represents a unique sequence, and each edge represents the pairwise connection between two sequences with an identity higher than 60%. GtrB-like sequences are included for comparison. (B) The active site of GtrB (PDB 5EKE). Catalytically essential residues are represented by cyan sticks. The Mg^2+^ cation and UDP molecule are represented with a green sphere and purple sticks, respectively. (C) WebLogo analysis of *pmrF*-like genes from (A) Conserved essential residues, such as Mg^2+^-binding site (DxD in residues 94 to 96 in GtrB numbers), two UndP-binding sites (R122 and R200), catalytic residue (D157), and UPG-binding sites [(Px(Y|F) and (F|Y)G(Q|K) as residues 10 to 12 and 72 to 74, respectively] are highlighted with dashed lines.

The sequence network analysis was conducted with the homologous *pmrF* and *GtrB* genes collected from the bacterial genomes and metagenomes. At least four discrete groups of the *pmrF* genes were clustered (Fig. 2A). Weblogo analysis of individually clustered genes revealed at least six sequence motifs highly conserved (Fig. 2B and C; Fig. S4 to 7), and they were tentatively assigned as Mg^2+^-binding site (DxD), UndP-binding site (R122 and R200 in GtrB numbering), and UDP-glucose-binding site [Px(Y|F) and (F|Y)G(Q|K)] (42). Notably, catalytic aspartate, which functions as a Lewis acid in GtrB (D157), was not observed in PmrF1 to PmrF3, although there was a conserved aspartate at the −4 position (Fig. 2C). Alternatively, they might not require such an acidic residue for glycosyltransferase activity, as suggested for the dolichylphosphate mannose synthase from Pyrococcus furiosus (*Pf*DPMS) (43); *Pf*DPMS and GtrB showed similar structures and sequence motifs except D157 in GtrB, and *Pf*DPMS has no acidic residue nearby the active site.

### Expression, isolation, and structural analysis of the *pmrE* and *pmrF* genes.

Putative PmrE and PmrF proteins were prepared using heterologous expression in E. coli (Fig. S8 to 12). After purification, we validated the oligomeric states of PmrE1 to PmrE4 and PmrF1 to PmrF3 by size exclusion chromatography; it was reported that substrate/product binding or mutations of UGDHs (36, 44) may alter oligomeric states and induce substantial conformational changes, suggesting that structural features of PmrE proteins might govern their biochemical functions.

The retention time and elution volume of PmrE1 to PmrE4 suggest that they are all tetramers (Fig. S9 and 10), indicating that they are different from hexameric UGDH from Homo sapiens (PDB 2Q3E) (45) and Caenorhabditis elegans (PDB 6OM8) (46) and dimeric UGDH from Pyrobaculum islandicum *DSM* 4184 (PDB 3VTF) (47), Burkholderia cepacia (PDB 2Y0E) (41), and Klebsiella pneumonia (PDB 3PID) (Fig. S13 and Table S3). These results are consistent with the sequence analysis that PmrE1 to PmrE4 proteins possess the protein-protein interactions (PPI) domain for dimerization but not hexamerization (Table S3, Fig. S14). PmrF1 to PmrF3 were also identified to be tetramers, resembling the oligomeric states of GtrB (42). These data were also consistent with the presence of PPI domains for tetramerization in the sequence analysis (Fig. S15).

### *In vitro* activities of the PmrE and PmrF proteins.

The steady-state activity of PmrE1 to PmrE4 was measured by altering the concentrations of NAD^+^ or UDP-glucose and monitoring time-dependent absorption changes at 340 nm. Then, Michaelis–Menten kinetic parameters of PmrE1 to PmrE4 were obtained from nonlinear iterative analysis (Fig. S16 and 17).

**FIG 3:**
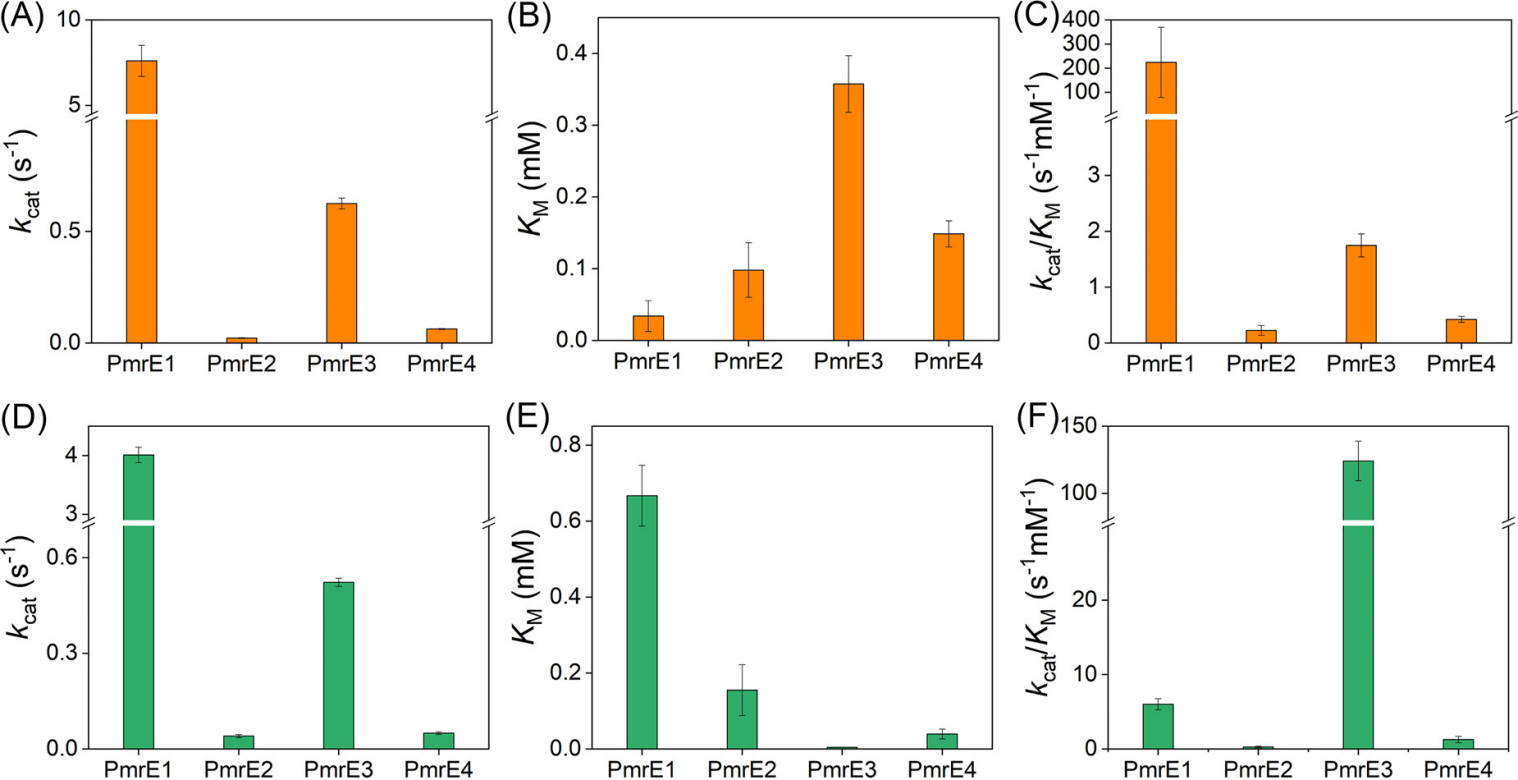
Michaelis-Menten kinetic parameters of the PmrE1 to PmrE4 proteins. The concentration of UPG (A–C) and NAD^+^ (D–F) are fixed as 2 and 3 mM, respectively. (A, D) *k*_cat_, (B, E) *K*_M_, and (C, F) *k*_cat_/*K*_M_ values are shown. All measurements were performed in triplicate.

All four PmrE proteins facilitated the reduction of NAD^+^ to NADH in the presence of UDP-glucose (Fig. 3A to C and Table 1). Although all discovered PmrE proteins (PmrE2 to PmrE4) exhibited considerably lower activity than PmrE1, they are kinetically competent in UDP-glucose oxidation coupled with NAD^+^ reduction. They showed discrete kinetic parameters determined with NAD^+^ (0 to 3 mM) and UDP-glucose (2 mM): PmrE1 ≫ PmrE3 > PmrE4 ≈ PmrE2 in turnover rates (*k*_cat_) and catalytic efficiencies (*k*_cat_/*K*_M_) and PmrE3 > PmrE4 ≈ PmrE2 > PmrE1 in the Michaelis constant (*K*_M_) for NAD^+^. Notably, PmrE3 showed a substantially high *K*_M_ value, suggesting that it may show a weak binding affinity for NAD^+^, and it could be related to sequence variations in one of the NAD^+^-binding regions [I(A|S)(V|T)(G|P)T(P|D)] different from others.

**TABLE 1:** The Michaelis-Menten kinetic parameters of PmrE1 to PmrE4

Protein	*k*_cat_ (s^−1^)	*K*_M_ (μM)	*k*_cat_/*K*_M_ (s^−1^ μM^−1^)
2 mM UDP-glucse/0–3 mM NAD^+^			
PmrE1	7.6 (0.9)	34 (22)	2.2 (1.5) × 10^−1^
PmrE2	2.2 (0.1) × 10^−2^	98 (38)	2.2 (0.9) × 10^−4^
PmrE3	6.2 (0.2) × 10^−1^	3.6 (0.4) × 10^2^	1.7 (0.2) × 10^−3^
PmrE4	6.3 (0.2) × 10^−2^	1.5 (0.2) × 10^2^	4.2 (0.5) × 10^−4^
3 mM NAD^+^/0–2 mM UDP-glucose			
PmrE1	4.0 (0.1)	6.7 (0.8) × 10^2^	6.0 (0.7) × 10^−3^
PmrE2	4.0 (0.5) × 10^−2^	1.5 (0.7) × 10^2^	2.6 (1.1) × 10^−4^
PmrE3	5.2 (0.1) × 10^−1^	4.2 (0.5)	1.2 (0.1) × 10^−1^
PmrE4	5.0 (0.4) × 10^−2^	39 (13)	1.3 (0.4) × 10^−3^

The order of reactivities of PmrE proteins was similarly detected when UDP-glucose concentration was varied (0 to 2 mM) with a fixed concentration of NAD^+^ (3 mM); PmrE1 ≫ PmrE3 > PmrE4 ≈ PmrE2 and PmrE3 ≫ PmrE1 > PmrE4 ≈ PmrE2, for *k*_cat_ and *k*_cat_/*K*_M_ values, respectively, when *K*_M_ values for UDP-glucose were determined in the following order: PmrE1> PmrE2 > PmrE4 > PmrE3 (Fig. 3D to F and Table 1). Notably, PmrE3 exhibited substantially lower *K*_M_ and higher *k*_cat_/*K*_M_ values than PmrE1 when PmrE1 showed relatively high *K*_M_ values. Because they possess highly conserved sequences that dictate UDP-glucose binding sites, dynamic motions that occur during the consumption of two substrates might determine their discrete reactivity. In addition, all Michaelis-Menten plots of PmrE1 to PmrE4 displayed nearly hyperbolic curves (Fig. S16 and 17), suggesting that two substrate-binding events occur noncooperatively without significant allosteric transition. These results contrasted with hexameric UGDH (44, 46), suggesting that discrete oligomerization states, tetramer versus hexamer, determine the mode of interaction with two substrates.

The proposed mechanism of UGDHs suggests that two sequential hydride transfers from UDP-glucose to NAD^+^ proceed via a nucleophilic cysteine residue (C253). The reaction was assisted by highly conserved residues, such as Y10, T118, K197, N201, K256, and D257 (the sequence numbers from UGDH in Klebsiella pneumonia) (41, 45, 48). These key residues were highly conserved in PmrE1 to PmrE4, indicating that residues other than those in the active sites were responsible for their discrete catalytic activity. Nevertheless, the catalytic activities of PmrE2 to PmrE4 demonstrated that they are kinetically competent UDP-glucose 6-dehydrogenases, and therefore, can be involved in lipid A modification for polymyxin resistance.

**FIG 4:**
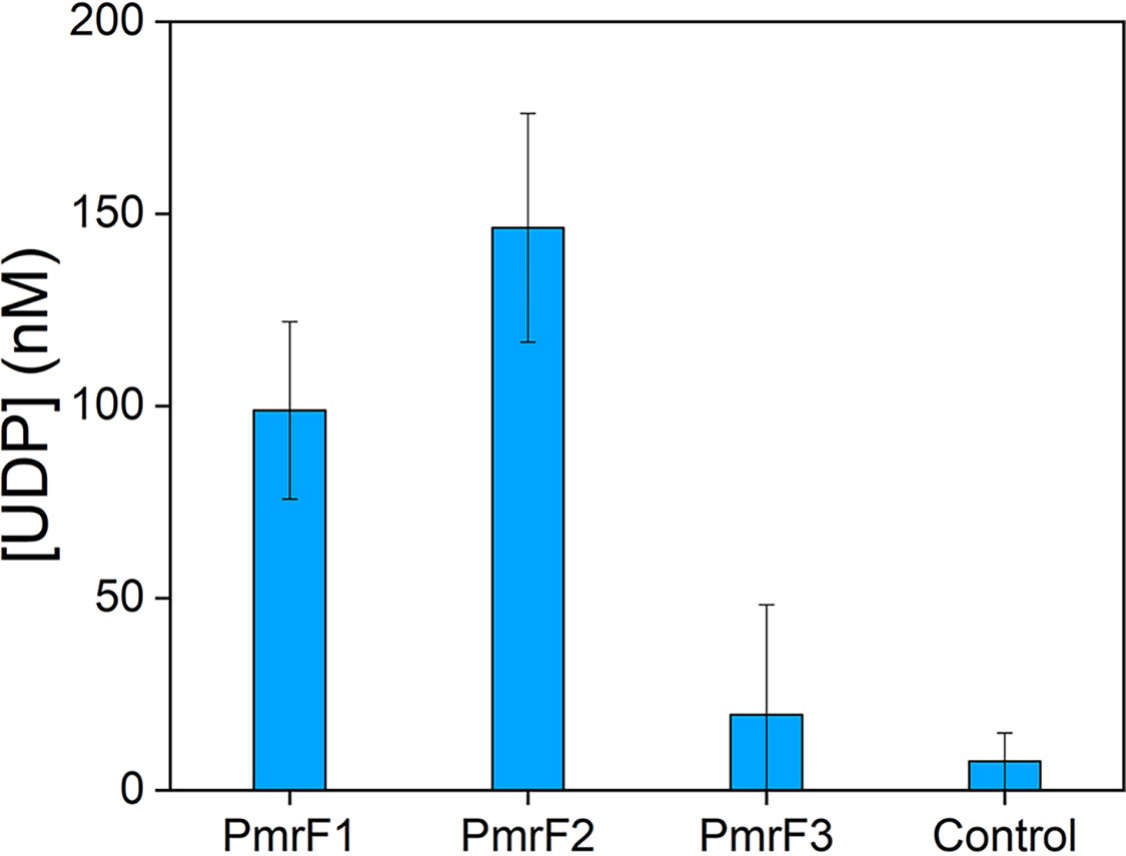
Glycosyltransferase activity of PmrF1, PmrF2, and PmrF3. The activity was measured with UndP (0.863 μM) and UPG (400 μM). An empty vector of pET-28b(+) is applied as a control. All measurements were performed in triplicate.

The activities of PmrF1 to PmrF3 with UDP-glucose and UndP were determined by measuring UDP concentration converted from UDP-glucose as a surrogate substrate for UDP-l-Ara4FN. Only PmrF1 and PmrF2 showed glycosyltransferase activity, but not with PmrF3 (Fig. 4), although they all possess sequence motifs that might be essential for substrate-binding. The lack of catalytic activity with PmrF3 might attribute to low protein stability because we observed that PmrF3 protein was aggregated during the assays and purification. Alternatively, unidentified residues critical for the reactivity may be absent in PmrF3. PmrF1 and PmrF2 yielded 99 ± 23 and 146 ± 30 nM of UDP, respectively, corresponding to 12 and 17% conversions of the added UndP (860 nM), respectively. The activity of PmrF1 and PmrF2 are lower than those of GtrB reported previously (up to 200 nM product formation) (42), possibly because UDP-glucose is not the native substrate for PmrF. Nevertheless, the presence of the catalytic activities of PmrF1 and PmrF2 suggest that they can participate in lipid A modification, possibly leading to polymyxin resistance.

### MICs of the discovered *pmrE* and *pmrF* genes.

**FIG 5:**
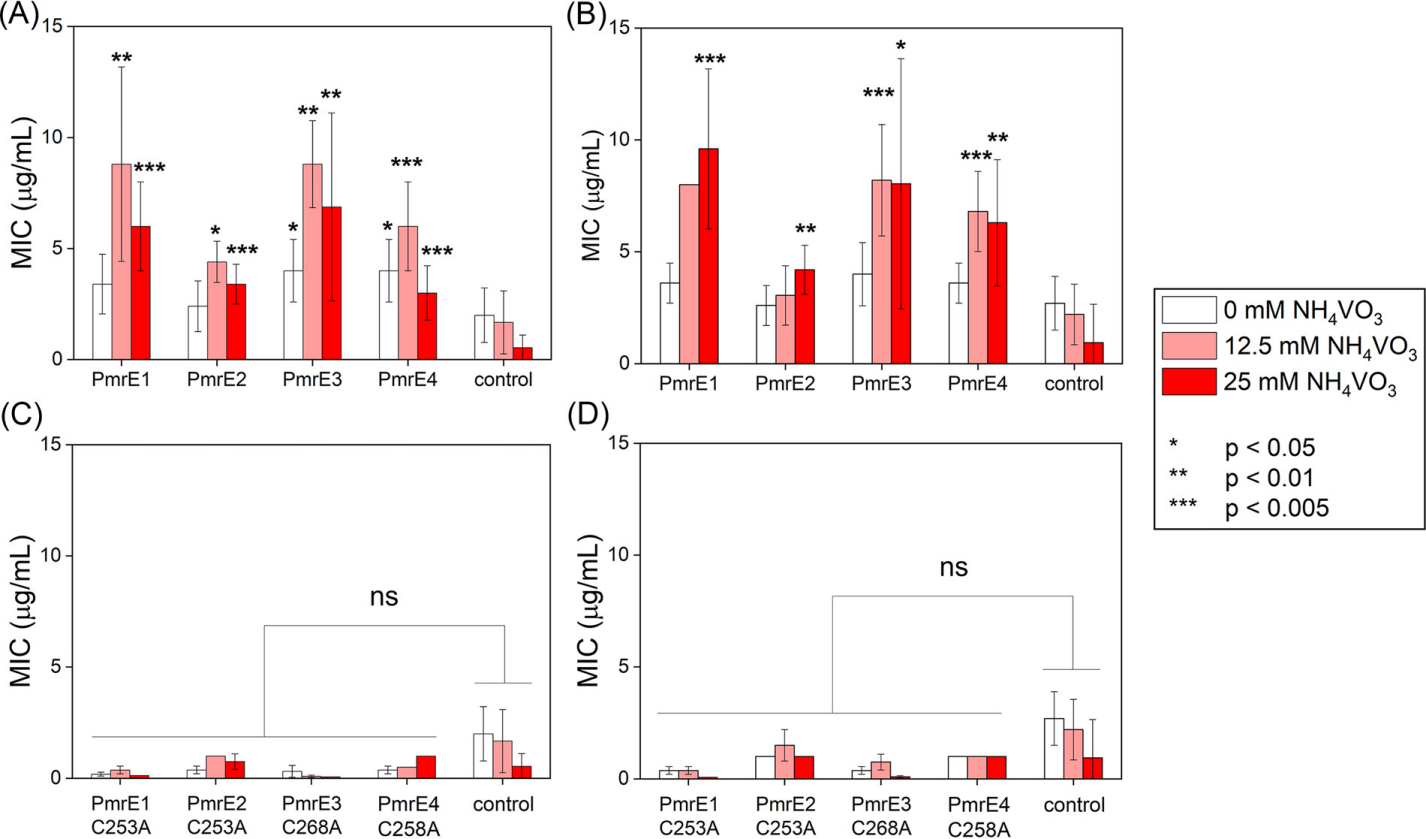
MIC values of PmrE1 to PmrE4 with polymyxin B2 and E upon different vanadate concentrations. PmrE1 to PmrE4 with (A) polymyxin B2 and (B) polymyxin E. Catalytically inactive single variants with (C) polymyxin B2 and (D) polymyxin E. An empty vector of pET-28b(+) is applied as a control. The error bars indicate the standard deviations of the five runs of the experiments.

To monitor whether the catalytic activities of the *pmrE* and *pmrF* genes detected under *in vitro* conditions are related to the development of polymyxin resistance, we measured the MICs. Two representative polymyxins, polymyxin B2 and E, were serially diluted in E. coli BL21(DE3) cells expressing the putative *pmrE* genes (Fig. 5A and B, white bars). Their MIC values are indistinguishable from those of the cells with empty pET vectors (control), presumably because *pmrE* genes alone are incompetent in lipid A modification. Thus, the coexpression of a series of other genes (Fig. S1C) is necessary to validate the biological activity of the isolated *pmrE* or *pmrF* genes.

E. coli K12 possesses an *arn* operon associated with lipid A modification that is dormant under normal cell growth conditions. The introduction of environmental stimuli, such as low Mg^2+^ or Ca^2+^ concentrations, low pH, osmotic shock, and high concentrations of metal ions, including Fe^3+^, Al^3+^, and metavanadate (VO_3_^−^) (12, 13), can activate the gene cluster, resulting in lipid A modification. Therefore, we applied one of these conditions to stimulate the gene cluster of E. coli BL21(DE3) and detect any significant increase in MIC values due to the heterologous expression of discovered *pmrE* genes.

When we added ammonium metavanadate (NH_4_VO_3_), the MIC values of cells overexpressing PmrE1 to PmrE4 proteins were substantially elevated with both polymyxins (Fig. 5A and B, red bars). PmrE2 to PmrE4 show considerably higher MIC values than those of the control with 12.5 mM or 25 mM NH_4_VO_3_. Their MIC values roughly correlated with the kinetic parameters of PmrE1 to PmrE4 measured under *in vitro* conditions (PmrE1 ≈ PmrE3 ≥ PmrE4 > PmrE2), indicating that polymyxin resistance is developed from the PmrE1 to PmrE4 proteins.

To further validate the *in vivo* activities of PmrE proteins, we prepared single variants in which a catalytic cysteine residue was mutated to alanine (Fig. 5C and D). The vanadate-dependent MIC values disappeared for all PmrE1 to PmrE4 variants, indicating that the polymyxin resistance observed above was derived from the catalytic activities of PmrE1 to PmrE4 in UDP-glucose oxidation, and the discovered *pmrE* genes contribute to polymyxin resistance.

**FIG 6:**
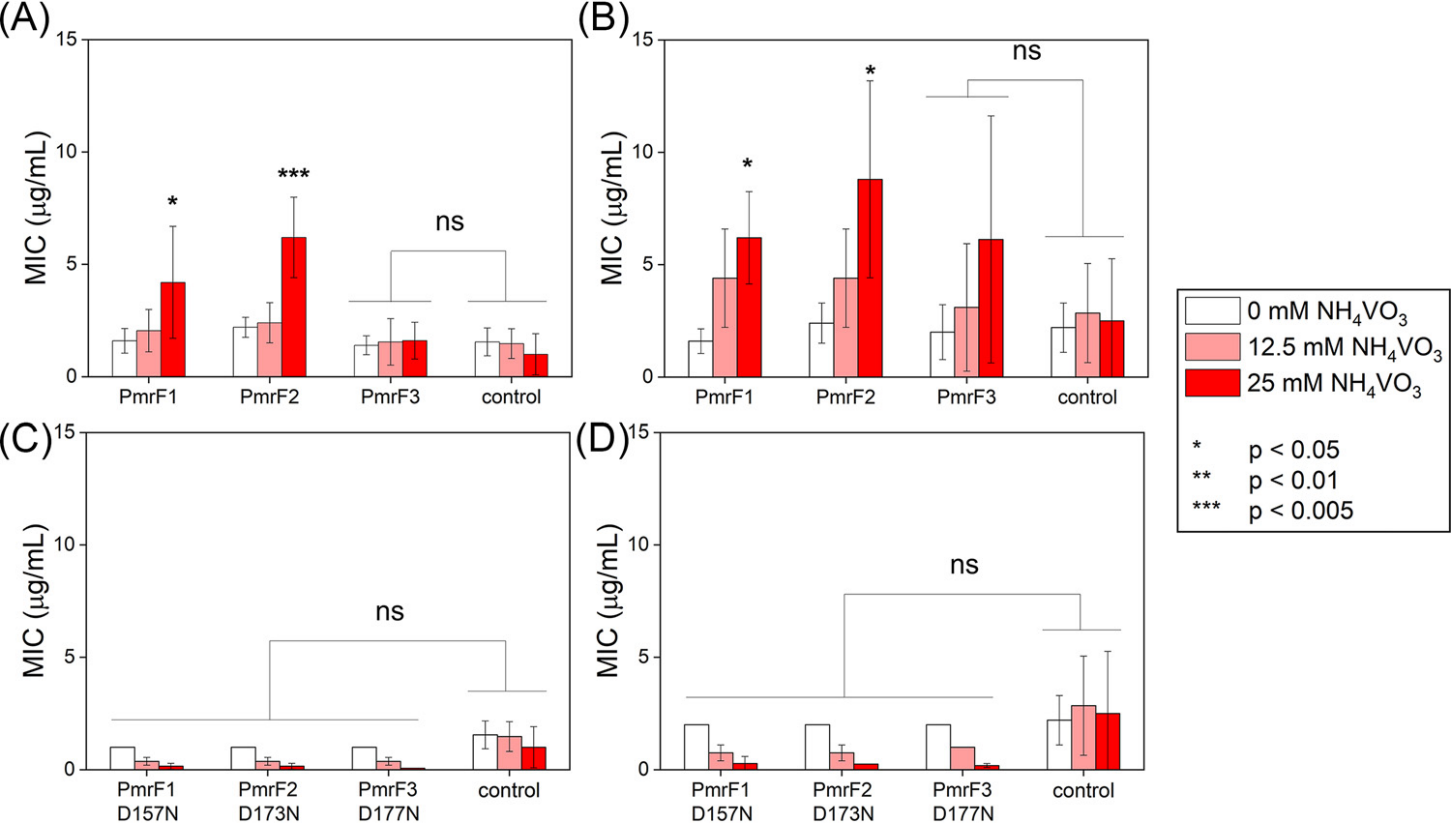
MIC values of PmrF1 to PmrF3 with polymyxin B2 and E upon different vanadate concentrations. PmrF1 to PmrF3 with (A) polymyxin B2 and (B) polymyxin E. Catalytically inactive single variants with (C) polymyxin B2 and (D) polymyxin E. An empty vector of pET-21b(+) is applied as a control. The error bars indicate the standard deviations of the five runs of the experiments.

We also measured the MIC values of BL21(DE3) pLysS cells expressing PmrF1 to PmrE3 proteins (Fig. 6A and B) with polymyxin B2 and E in the absence and presence of NH_4_VO_3_. The MIC values of PmrF1 and PmrF2 were detected only in 25 mM NH_4_VO_3_ for both polymyxins but not PmrF3 regardless of NH_4_VO_3_ concentration. These results were consistent with the catalytic activities observed under *in vitro* conditions, indicating that *pmrF*2 gene discovered from sediment and seawater microbiome can induce polymyxin resistance, but not *pmrF3*.

Upon mutation of the conserved acidic residue, which may correspond to D157 in GtrB in the sequence alignments (Fig. 2C), into asparagine, the effective MIC values of PmrF1 and PmrF2 disappeared (Fig. 6C and D). These results indicate that the MIC values observed above were indeed derived from the reactivities of PmrF1 and PmrF2. The data also suggest that the conserved aspartate residue plays a critical role in the transferase activity, similarly to D157 in GtrB, and is an essential sequence motif that dictates the chemical function of *pmrF* gene.

### Conclusion.

We discovered and characterized *pmrE* and *pmrF* genes from metagenomes under *in vitro* and *in vivo* conditions and compared them with those from E. coli. Three *pmrE* genes (*pmrE2* to *pmrE4*) and one *pmrF* gene (*pmrF2*) displayed reactivity essential in lipid A modification, suggesting that their activities were directly related to the emergence of polymyxin resistance. In particular, whereas *pmrE* genes have been extensively investigated, the discovered *pmrE* genes are considerably dissimilar from others. In addition, we directed measured *in vitro* activities of *pmrF* genes, including the one from E. coli. Site-directed mutagenesis studies of *pmrF* genes also indicate that they require an acidic residue for transferase activity. This work demonstrated that *pmrE* and *pmrF* genes exhibit diverse sequence and function, expanding polymyxin resistomes.

## MATERIALS AND METHODS

### Data collection.

We have obtained 2,557 putative *pmr* genes from sediment and seawater samples using homology search against the CARD database (40% ≤ sequence similarity < 80% and query coverage ≥ 70%). After clustering the sequences with a threshold of 40% sequence similarity, five nonredundant *pmr* genes were retained from each cluster that had more than five *pmr* genes (*pmrE2* to *pmrE4* and *pmrF2* to *pmrF3*; see supporting information in Table S1). Additionally, two *pmr* genes (*pmrE1* and *pmrF1*) were obtained from the CARD database as a reference.

### Genome mining of putative PmrE proteins.

Position-specific iterative BLAST (PSI-BLAST) (49) was performed in December 2021 on reference protein database and metagenomic protein database using four putative PmrE1 to PmrE4 proteins with a cutoff value of 50% coverage and 25% sequence identity. A total of 4,004 proteins were used to generate a sequence-similarity network (SSN) using EFI-EST (http://efi.igb.illinois.edu/efi-est) (50). The resulting network was visualized in Cytoscape 3.8.1 using organic layout (Fig. S2) (51).

### Sequence and structure analysis.

For structure-guided sequence analysis of PmrE-like proteins, we collected the sequences of nine UGDHs, of which X-ray crystal structures or oligomeric sizes were identified (Table S3). The sequence identity was obtained from the BLAST global alignments. Sequences included for alignments in Fig. 1 are as follows: Streptococcus pyogenes (UniProtKB P0C0F4), Burkholderia cepacia(C9E261), Klebsiella pneumoniae (A0A0J9WZA6), Pseudomonas aeruginosa PA2022 (GenBank accession number NP_250712), Pseudomonas aeruginosa PA3559 (NP_252249), Pyrobaculum islandicum (UniProtKB A1RUM9), *Sphingomonas elodea* (A4UTT2), Porphyromonas gingivalis (Q7MVC7), Caenorhabditis elegans (Q19905), and Homo sapiens (O60701). We conducted multiple sequence alignments using Clustal Omega (Fig. 1 and Fig. S3) (52). The NAD^+^ and UDP-glucose-binding motifs were defined by inspecting the residues that show direct contact with the bound substrates in the X-ray crystal structures and high degrees of conservation in the sequence alignments. To identify protein-protein interface (PPI) domains for dimer and hexamer formations, we inspected the X-ray crystal structures of UGDH from Klebsiella pneumoniae (PDB 3PLN) and Homo sapiens (PDB 4RJT), respectively. For structure-guided sequence analysis of PmrF-like proteins, GtrB (PDB 5EKP) structure was applied.

### Genome mining of putative PmrF proteins.

Position-specific iterative BLAST (PSI-BLAST) was performed in December 2021 on reference protein database and metagenomic protein database using three putative PmrF1 to PmrF3 proteins and GtrB protein with a cutoff value of 50% coverage and 25% sequence identity. A total of 3,491 proteins were used to generate an SSN using EFI-EST (http://efi.igb.illinois.edu/efi-est) (50). The resulting network was visualized in Cytoscape 3.8.1 using organic layout (Fig. 2A) (51). At least four groups (1–4) were identified, which includes PmrF1 to PmrF3 and GtrB, individually. The number of sequences for each network that include PmrF1, PmrF2, PmrF3, and GtrB is 541, 572, 958, and 543, respectively. The genes included within each group were aligned using MAFFT 7.490 with a G-ins-i algorithm option (53). The alignment was used to generate sequence logos using WebLogo3 (Fig. 2C and Fig. S4 to 7) (54).

### Expression and purification of PmrE proteins.

We selected *pmrE1*, *pmrE2*, *pmrE3*, and *pmrE4* as the target genes for biochemical characterization. Prior to gene synthesis, the codons of the DNA fragments were optimized for further E. coli expression (General Biosystems). The genes were cloned into pET28b(+)/kan^R^ vector using NdeI and XhoI restriction enzyme sites and transformed to either DH5α or BL21(DE3) for sequencing or protein expression, respectively. All protein sequences were followed by a six-histidine tag at the N terminus.

For the expression of target PmrE, picked a single colony of BL21(DE3) and inoculated in 10 mL autoclaved LB media containing 50 mg/L kanamycin. The cells were grown in a 200-rpm orbital shaker at 37°C for 18 h and inoculated in 1 L autoclaved LB media containing 50 mg/L kanamycin. At an optical density at 600 nm (OD_600_) value of 0.7, the temperature was reduced to 15°C and induced with 0.1 mM isopropyl-β-D-thiogalactopyranoside (IPTG). After growth in a 150-rpm orbital shaker for 18 h, the cells were harvested by centrifugation at 5,000 rpm for 10 min at 4°C and the cell pastes were frozen in liquid nitrogen and stored at −80°C for further usage.

The cell pastes were resuspended in lysis buffer (25 mM Tris/HCl buffer, pH 8.0) and lysed by sonication for 30 min in an ice bath (on/off = 3 s each). After centrifugation at 13,000 rpm for 30 min at 4°C, the supernatants were loaded to a Ni affinity column (HisTrap FF column, GE Healthcare Life Sciences), preequilibrated with the lysate buffer at 4°C by the ÄKTA protein purification system. Applying elution buffer (25 mM Tris/HCl buffer, pH 8.0 with 500 mM imidazole) in a linear gradient (5 to 50%) eluted all proteins around ~100 mM imidazole condition (Fig. S8). The relatively pure fractions (~80 to 90%) were determined by SDS-PAGE and concentrated using a centrifugal concentrator with 10-kDa cutoff membrane filters. The purification step was followed by size exclusion chromatography (HiLoad 16/600 Superdex 200 pg) with the buffer of 25 mM Tris/HCl buffer, pH 8.0 with 150 mM NaCl (Fig. S10).

The purified protein was concentrated up to ~10 to 100 μM and stored at −80°C until further usage. The protein concentration was determined by UV-Visible spectrophotometer (Agilent Cary 8454) using the absorption coefficients at 280 nm estimated from the sequence.

### Expression and purification of PmrF proteins.

We selected *pmrF1*, *pmrF2*, and *pmrF3* as the target genes for biochemical characterization. Prior to gene synthesis, the codons of the DNA fragments were optimized for further E. coli expression (General Biosystems). The genes were cloned into pET21b(+)/amp^R^ vector using NheI and XhoI restriction enzyme sites and transformed to either DH5α or C41 for sequencing or protein expression, respectively. All protein sequences were followed by a six-histidine tag at the N terminus.

For the expression of target PmrF, we picked a single colony of C41 and inoculated in 10 mL autoclaved LB media containing 100 mg/L ampicillin. The cells were grown in a 200-rpm orbital shaker at 37°C for 18 h and inoculated in 1 L autoclaved LB media containing 100 mg/L ampicillin. At an OD_600_ value of 0.7, the temperature was reduced to 22°C and induced with 0.5 mM IPTG. After grown in a 150-rpm orbital shaker for 18 h, the cells were harvested by centrifugation at 5,000 rpm for 10 min at 4°C and the cell pastes were frozen in liquid nitrogen and stored at −80°C for further usage.

The cell pastes were resuspended in lysis buffer (25 mM sodium HEPES buffer, pH 7.5 with 150 mM NaCl and 20 mM MgSO_4_) and lysed by sonication for 30 min in an ice bath (on/off = 3 s each). After centrifugation at 13,000 rpm for 30 min at 4°C, the pellet was resuspended in extraction buffer (lysis buffer with 1% [wt/vol] n-dodecyl-β-maltoside (DDM)). The resuspended mixture was incubated at 100 rpm for 1 h at 15°C. The mixtures were centrifuged at 13,000 rpm for 30 min at 4°C and the supernatants were loaded to a Ni affinity column (HisTrap FF column, GE Healthcare Life Sciences), preequilibrated with the extraction buffer at 4°C by the ÄKTA protein purification system. An elution buffer (extraction buffer with 500 mM imidazole) was applied in a linear gradient (5 to 50%), resulting in the elution of PmrF proteins (Fig. S11). The relatively pure fractions (~80 to 90%) were determined by SDS-PAGE and concentrated using centrifugal concentrator with 10-kDa cutoff membrane filters. The purification step was followed by size exclusion chromatography (HiLoad 16/600 Superdex 200 pg) with 25 mM sodium HEPES buffer (pH 7.5) with 150 mM NaCl and 0.1% (wt/vol) DDM (Fig. S12). The purified protein was concentrated up to ~10 to 100 μM and stored at −80°C until further usage. The protein concentration was determined by UV-Visible spectrophotometer (Agilent Cary 8454) using the absorption coefficients at 280 nm estimated from the sequence.

### *In vitro* activity assay of PmrE.

Two substrates, NAD^+^ and UDP-glucose, were dissolved in water, and diluted with the buffer used for the assay. Various concentrations of the substrates, NAD^+^ and UDP-glucose, were mixed with 1 μM protein in 400 μL of 50 mM Tris (pH 8.7) buffer with 1% (wt/vol) dithiothreitol (DTT). The initial rate was measured by detecting the concentrations of NADH formation by time-resolved absorption changes at 340 nm by UV-Visible spectrophotometer (Agilent Cary 8454). Steady-state kinetic parameters of PmrE proteins were obtained by varying the concentrations of one of the substrates, either NAD^+^ or UDP-glucose, when the other was fixed to be 3 mM or 2 mM, respectively. The kinetic parameters, *k*_cat_, *K*_M_, and *k*_cat_/*K*_M_, were determined from nonlinear iteration curve fit to the Michaelis-Menten equation (Table 1, Fig. 3, Fig. S16 and 17).

### *In vitro* activity assay of PmrF.

The biochemical function of PmrF proteins were determined by using the method of GtrB activity assay (42). In short, the *pmrF* genes were transformed into E. coli C41 competent cells. After cell growth, protein expression, the lysis of cell pellets (2 g), and centrifugation, as described above, the pellets were resuspended in 30 mL of 100 mM Tris/HCl pH 8.0 buffer and sonicated for 1 h in an ice bath. The resuspension (5 μL) was mixed with 250 μL of 100 mM Tris/HCl (pH 8.0) buffer containing 10 mM MgCl_2_ and 1 mM EDTA and 400 μM UPG and 0.86 μM UndP at the final concentrations. The solution was incubated for 1 h at room temperature after mild sonication for mixing. Then, the UDP-Glo glycosyltrasnferase assay kit (Promega) was used to measure the concentrations of UDP by luminescence. The standard curve of luminescence intensity versus UDP concentration was measured independently (Fig. S18). Protein concentration was quantified by densitometry of SDS-PAGE gel.

### Determination of MIC values.

MIC values for BL21(DE3) cells containing *pmrE* genes and BL21(DE3) pLysS cells containing *pmrF* genes were measured following the previously reported procedures (31). In short, the plasmids containing *pmrE* or *pmrF* genes were transformed into BL21(DE3) or BL21(DE3) pLysS cells, respectively. The cells were grown on the LB/agar plate containing 50 mg/L kanamycin or 100 mg/L ampicillin and 35 mg/L chloramphenicol overnight at 37°C. The cell culture was diluted with 0.85% saline until the OD_625_ reached 0.1, followed by mixing the 100 μL aliquot with 18.9 mL Mueller-Hinton broth. Polymyxin B2 and polymyxin E (0 to 128 μg/mL) were dissolved in deionized water and added. After incubating at 34°C overnight in a nontreated 96-well cell polystyrene culture microplate (SPL Life Sciences) (55, 56), optical cell density (OD_625_) was measured with the microplate reader (BioTeK Synergy H1) to determine the MICs of the antibiotics (Fig. 5 and 6). We conducted the experiments at least five times, and statistical analysis was performed using Student's *t* test.

### Data availability.

Additional data, including sequence information, protein purification, kinetic data, and MIC results, are provided in the supplementary materials.
