# Supplementary material for: Discovery and Characterization of Polymyxin-Resistance Genes pmrE and pmrF from Sediment and Seawater Microbiome
Source: Microbiol Spectr. 2023 Jan 5;11(1):e02736-22. doi: 10.1128/spectrum.02736-22 (PMC9927302; doi:10.1128/spectrum.02736-22)
Supplement: Supplemental file 1 — Supplemental material. Download spectrum.02736-22-s0001.pdf, PDF file, 6.3 MB [file spectrum.02736-22-s0001.pdf]

Supporting Information for

Discovery and Characterization of Polymyxin-Resistance Genes *pmrE* and *pmrF* from Sediment and Seawater Microbiome

Hwanjin Joo,[1] Hyunuk Eom,[1] Youna Cho,[2] Mina Rho,[2,3,]* Woon Ju Song[1,]*

[1]Department of Chemistry, Seoul National University, Seoul 08826, Republic of Korea
[2]Department of Computer Science, Hanyang University, Seoul, 04763, Korea
[3]Department of Biomedical Informatics, Hanyang University, Seoul 04763, Republic of Korea.
*Email: minarho@hanyang.ac.kr, woonjusong@snu.ac.kr

Table S1. The sequence information. (a) Sediment and seawater microbial samples (b) PmrE1–4 (c) PmrF1–3

(a)

Gene	Sample	Accession Number	Site	Latitude	Longitude	Read count	File size (G)
pmrE2	SKY4B1505	ERR2092769	Korean ocean	34°45′50″N	127°48'00″E	347,769,806	116
pmrE3	IO2	ERS489087	Indian ocean	6°00'00.4"N	73°53'43.8"E	366,582,722	84
pmrE4	SKY2B1505	ERR2092767	Korean ocean	34°57′55″N	127°45′40″E	392,703,700	130
pmrF2	SR-TA-A-1909	ERR7460768	Korean ocean	36°53'50.698"N	126°17'35.138"E	81,750,158	30
pmrF3	NH3	ERR2431953	Korean sediment	34°79′01″N	127°56′55″E	253,427,114	84

(b)

Protein	Sequence
PmrE1	MKITISGTGYVGLSNGLLIAQNHEVVALDILPSRVAMLNDRISPIVDKEIQQFLQSDKIHFNATLDKN EAYRDADYVIIATPTDYDPKTNYFNTSSVESVIKDVVEINPYAVMVIKSTVPVGFTAAMHKKYRTE NIIFSPEFLREGKALYDNLHPSRIVIGERSERAERFAALLQEGAIKQNIPMLFTDSTEAEAIKLFANTYL AMRVAYFNELDSYAESLGLNSRQIIEGVCLDPRIGNHYNNPSFGYGGYCLPKDTKQLLANYQSVPN NLISAIVDANRTRKDFIADAILSRKPQVVGIYRLIMKSGSDNFRASSIQGIMKRIKAKGVEVIIYEPVM KEDSFFNSRLERDLATFKQQADVIISNRMAEELKDVADKVYTRDLFGSD
PmrE2	MKITVAGLGYVGLSNAVLLAQNHTVTAIDISQDRVDQVNAKTSPIVDADIEDFLANHTLDLTATTD AEAAYKDADFIIVATPTNYDAQSNYFDTSSVETVINHALKANPNATIIVKSTIPVGFIDGIRTQMNSQ NIVFSPEFLREGRALYDNLHPSRIIVGAQTEAAKTFANLLIEGAITKDVLVQFTDASEAEAIKLFANT YLAMRVAFFNELDSYAMSRGMDSRQIINGISLDPRIGNHYNNPSFGYGGYCLPKDTKQLLANYSEV PQNLIRAIVDANRTRKDFLSDRIIAMQPNIVGVHRLVMKAGSDNFRQSSIQGIMKRVKAKGIEVIVY EPELQETEFFNSRVITDLEAFKAEADVIVANRITDDLRDVAAKVFSRDLFGAD
PmrE3	MKQLGINEMNPGLPKQICIIGAGYVGMSYAVLISSFADIKIWDIDSKKRDLINAKKLPIQDLDSESILS EKENWNIVASKNLNEALNKSQLVLICISTDFNESKNSFDVNEMNNLIDQVRKYSPNVQIVIKSTVPIG YSAKITQETGLNILFSPEFLREGMAIRDNQFPSRIIIGKTNQNQACDPYLSVAKEIAKNSPEIFEMSASE AEAVKLFSNSYLAMRIAFFNEVDGFALKNNLLIKDIIEGMSADNRIGNYYNNPSFGFGGYCLPKDSR QALVSMNDLPNEIIQSINISNSKRKEFISKYLLHMDKDLYGFYRINMKENSDNMRESASIEIIKILLSA GKQVIIYEPLLNNTNDFDNFELVKNLDEFKERSDIIIANRVTEEILDCKEKLFSRDLSYDTKIRPKNI
PmrE4	MLNKKVLVFGAGYVGFSLSVVMARAANVTVVDIRPDIIRSINAGRSPIEDLDIDKHLMIGLSSNRLN AQLYSQKLIEEADFVVLALPTSFNPEVAGFDTSALDDVIAKVADIDKSKPIIIKSTIPVGYTQKIIEKFG LSECYYSPEFLREGRATYDNLNPSRIVIGSTSTHAKEFVKILDDASHQRNTKKVFTDNTTAEVIKLAA NSYLAARVSYFNELDTLAMIAGLNAVQLIDGVCADPRIGDGYNNPSFGYGGYCLPKDVKQFQRSFL DFKIHAPLIQSIDASNQQRIVEIINFVKSSGAKNIGIYRAQMKQGSDNARDSVNLAVLSQLSAMPTLR VKIFEPKIDLPENLSTFKVNEFETFCDWSDLILANRDAVELREYHYKVLTRDIYNEN

(c)

Protein	Sequence
PmrF1	MFEIHPVKKVSVVIPVYNEQESLPELIRRTTTACESLGKEYEILLIDDGSSDNSAHMLVEASQAENS HIVSILLNRNYGQHSAIMAGFSHVTGDLIITLDADLQNPPEEIPRLVAKADEGYDVVGTVRQNRQD SWFRKTASKMINRLIQRTTGKAMGDYGCMLRAYRRHIVDAMLHCHERSTFIPILANIFARRAIEIPV HHAEREFGESKYSFMRLINLMYDLVTCLTTTPLRMLSLLGSIIAIGGFSIAVLLVILRLTFGPQWAAE GVFMLFAVLFTFIGAQFIGMGLLGEYIGRIYTDVRARPRYFVQQVIRPSSKENE

PmrF2	MISYLSSVIIAQLQNPQVKNTMLDLSVIIPIYNEQDSIPELYQRTHETLEKLGRSYEIIFVNDGSADKS AILLDELHEQDSQHVKVIHFNGNFGQHMAIMAGFENSTGLAVVTLDADLQNPPEEIPKLITAMDEG HDIVEGMRQARKDNAFRRYASRLNNWIRHKTTGIRLKDQGSMLRAYNRRVVELMVLSKERATYI PALAYSYASNPGFVEVNHAERAHGESKYSLFRLLRLHFDLMAGFSSAPLQFVTLTGMGISFFSFIFF IFMVLRRIIVGPEVQGVFSLFALLFLILGFLIFAVGLVGEYVGRIYLEVRNRPRFVIRKILEPSKITAA KTPKTKQEKQINTKKAEKPGEESPPKTE
PmrF3	MSDGMMDLTNFHPETDVFAPIQTTNKVDVSVVIPVFNEDESIPELHNRLTTSLLSTGKNYEIIYIDD GSTDGSFEKLKSIQYQDSRVWIIQLRRNFGQAAAFSAGFDLAHGEVIVTLDGDLQNDPADIPNLLE KLDEGFDVVSGWRVNRKDQFLTRRVPSILANAMISRVTGLELHDYGCSLKAYRQEVVKNIKLYG ELHRFIPAIASWMGIKVAEIPVNHAPRKHGRSHYGLGRTLKVFLDLITVKFLLNYATRPLQIFGLAG MLSFVAGMGLSIYLTILRLFFNQPLSNRPILLLAILLIMLGVQLIVMGLLGELIVRTYHESQGKSIYV VRNVLHSPDGSKQES

Table S2. Sequence identity value compared to each other sequence for (a) *pmrE*1-4 and (b) *pmrF*1-3. All sequence identity values were calculated by BLAST global alignments.

(a)

	*pmrE*1	*pmrE*2	*pmrE*3	*pmrE*4
*pmrE*1	-	64%	39%	41%
*pmrE*2	-	-	39%	39%
*pmrE*3	-	-	-	34%
*pmrE*4	-	-	-	-

(b)

	*pmrF*1	*pmrF*2	*pmrF*3
*pmrF*1	-	41%	40%
*pmrF*2	-	-	34%
*pmrF*3	-	-	-

Table S3. The oligomeric state of various UGDH proteins.

Oligomeric State	PDB code	Organism (UniprotKB ID or GeneBank™ accession number)	Reference
Monomer	-	*Streptococcus pyogenes* (P0C0F4)	(1)
Dimer	2Y0E	*Burkholderia cepacia* (C9E261)	(2)
	-	*Escherichia coli* K-5	(3)
	3PLN	*Klebsiella pneumonia* (A0A0J9WZA6)	(4)
	-	*Pseudomonas aeruginosa* (NP_250712/252249)	(5)
	3VTF	*Pyrobaculum islandicum* (A1RUM9)	(6)
	4A7P	*Sphingomonas elodea* (A4UTT2)	(7)
	7KWS	*Campylobacter jejuni NCTC 11168*	(8)
Tetramer	3GG2	*Porphyromonas gingivalis* (Q7MVC7)	Unpublished§
	-	*Escherichia coli* (PmrE1)	This work
	-	Metagenome (PmrE2)	This work
	-	Metagenome (PmrE3)	This work
	-	Metagenome (PmrE4)	This work
Hexamer	2O3J	*Caenorhabditis elegans* (Q19905)	unpublished§
	2Q3E/4RJT	*Homo sapiens* (O60701)	(9, 10)
Dodecamer	-	*Halobacterium salinarum*	(11)
	-	*Haloferax volcanii*	(11)

§The biological assembly was assigned by authors and generated by PISA (software).

Table S4. Kinetic parameters of various PmrE from different organisms for various concentrations of (a) NAD^+ (b) UDP-glucose. The assays were conducted with 50–100 mM Tris/HCl (pH 8.7) buffer with minor alterations as noted in the last column.

(a) NAD^+ with 2 mM UDP-glucose

Organism	k_{cat} (s^{-1})	K_M (mM)	k_{cat}/K_M ($mM^{-1}s^{-1}$)	Ref	Condition
Streptococcus pyogenes	1.8(0.1)	0.065(0.006)	27(3)	(12)	2 mM DTT
Burkholderia cepacia	6.7	0.53	12	(2)	10 mM $MgCl_2$
Klebsiella pneumoniae	0.037(0.003)	0.11(0.01)	0.066(0.006)	(13)	1 mM DTT
Sphingomonas elodea	7.63	0.4	19	(14)	5 mM DTT/ 10 mM $MgCl_2$
Sphingomonas sanxanigenens	0.84	0.38	2.2	(15)	5 mM DTT/ 10 mM $MgCl_2$
Escherichia coli (PmrE1)<br>Metagenome (PmrE2)<br>Metagenome (PmrE3)<br>Metagenome (PmrE4)	7.6(0.9)<br>2.2(0.1) x 10^{-2}<br>6.2(0.2) x 10^{-1}<br>6.3(0.2) x 10^{-2}	0.034(0.022)<br>0.098(0.038)<br>0.36(0.04)<br>0.15(0.02)	2.2(1.4) x 10^{2}<br>0.22(0.09)<br>1.7(0.2)<br>0.42(0.05)	This work	1 mM DTT

ND: Not determined

(b) UDP-glucose with 3 mM NAD^+

Organism	k_{cat} (s^{-1})	K_M (mM)	k_{cat}/K_M (mM^{-1} s^{-1})	Ref	condition
Streptococcus pyogenes	1.8(0.1)	2.0(0.4) x 10^{-2}	90(17)	(12)	2 mM DTT
Burkholderia cepacia	6.9	0.23	30	(2)	10 mM $MgCl_2$
Klebsiella pneumoniae	0.037(0.003)	0.67(0.03)	0.011(0.001)	(13)	1 mM DTT
Sphingomonas elodea	8.7	0.87	10	(14)	5 mM DTT/ 10 mM $MgCl_2$
Sphingomonas sanxanigenens	0.97	0.47	2.1	(15)	5 mM DTT/ 10 mM $MgCl_2$
Escherichia coli (PmrE1)<br>Metagenome (PmrE2)<br>Metagenome (PmrE3)<br>Metagenome (PmrE4)	4.0(0.1)<br>4.0(0.5) x 10^{-2}<br>5.2(0.1) x 10^{-1}<br>5.0(0.4) x 10^{-2}	0.67(008)<br>0.15(0.07)<br>4.2(0.5) x 10^{-3}<br>0.039(0.013)	6.0(0.7)<br>0.26(0.12)<br>12(1) x 10^{2}<br>1.3(0.4)	This work	1 mM DTT

Table S5. The MIC values of polymyxin-resistant bacteria.

Organism	MIC (µg/mL)		Reference
	Polymyxin B	Polymyxin E (colistin)	
Acinetobacter baumannii	ND	3–10	(16)
Salmonella enterica YL14P053	ND	4	(17)
Cronobacter sakazakii WF5-21C	ND	4	(18)
Pseudomonas aeruginosa	0.5–8	ND	(19)
Escherichia coli EC1002	4	4	(20)
Escherichia coli (PmrE1)	>4*	8*	This work
Metagenome (PmrE2)	>2*	>4*	This work
Metagenome (PmrE3)	>4*	>8*	This work
Metagenome (PmrE4)	>4*	>4*	This work
Escherichia coli (PmrF1)	>2*	>4*	This work
Metagenome (PmrF2)	>4*	>4*	This work
Enterobacter aerogenes	8	4	(21)
Enterobacter cloacae GB38	>32	>32	(22)
Burkholderia multivorans ATCC 17616	256	512	(23)
Serratia marcescens 3927	2,048	ND	(24)

ND: Not determined
*Measured in the presence of 25 mM NH_4VO_3

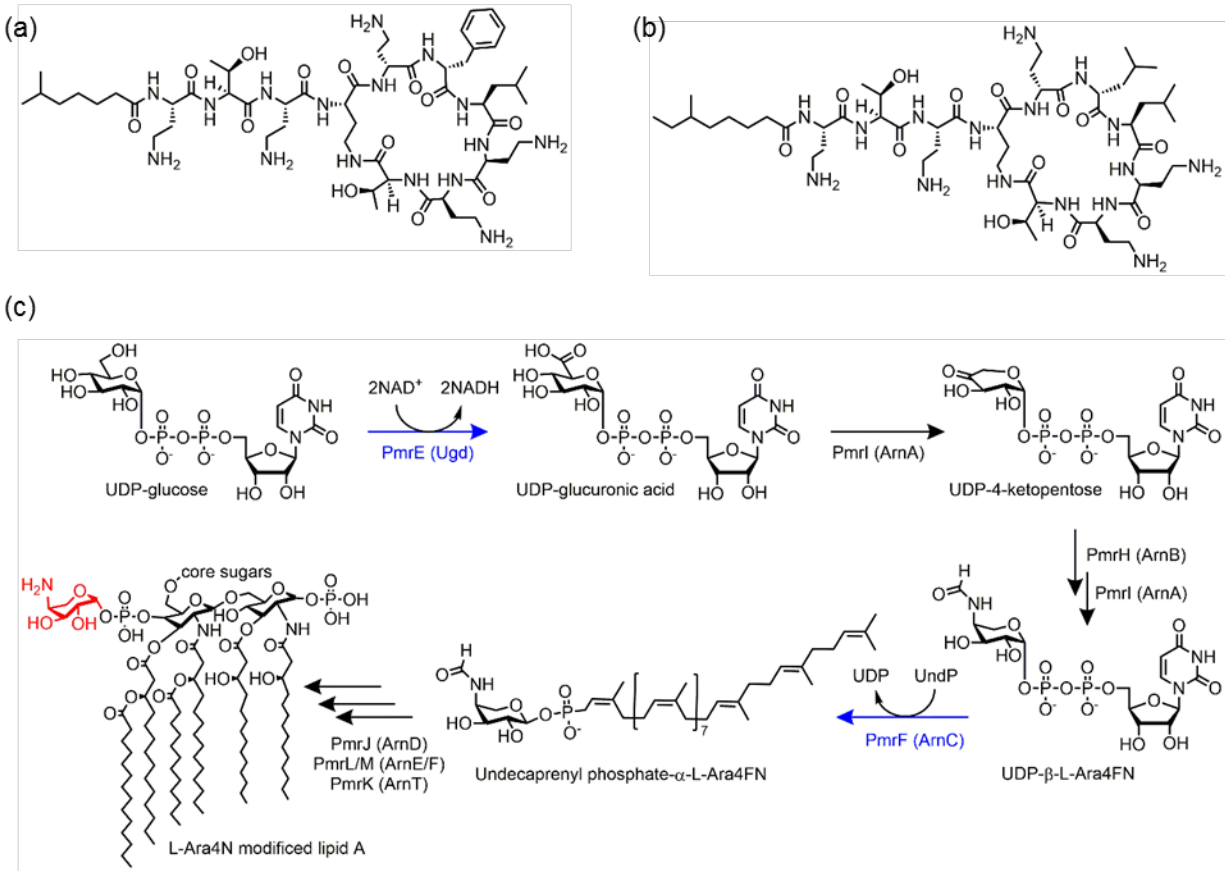

Figure S1. Polymyxin and its antibiotic resistance. The molecular structure of (a) polymyxin B2 and (b) polymyxin E. (c) The modification pathway of Lipid A. L-Ara4N moiety is highlighted in red. The reactions mediated by the genes addressed in this work are highlighted in blue.

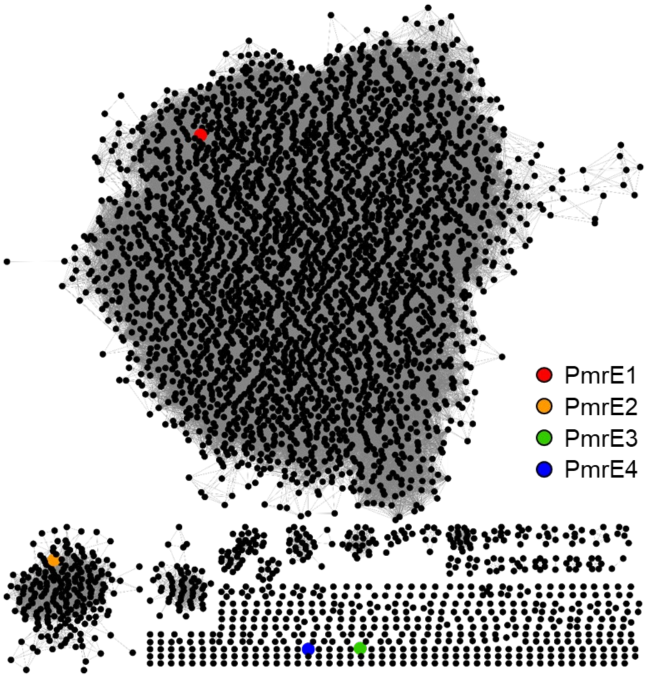

Figure S2. The sequence similarity network analysis of PmrE-like proteins. Each node represents a unique sequence, and each edge represents the pairwise connection between two sequences with sequence identity higher than 80%.

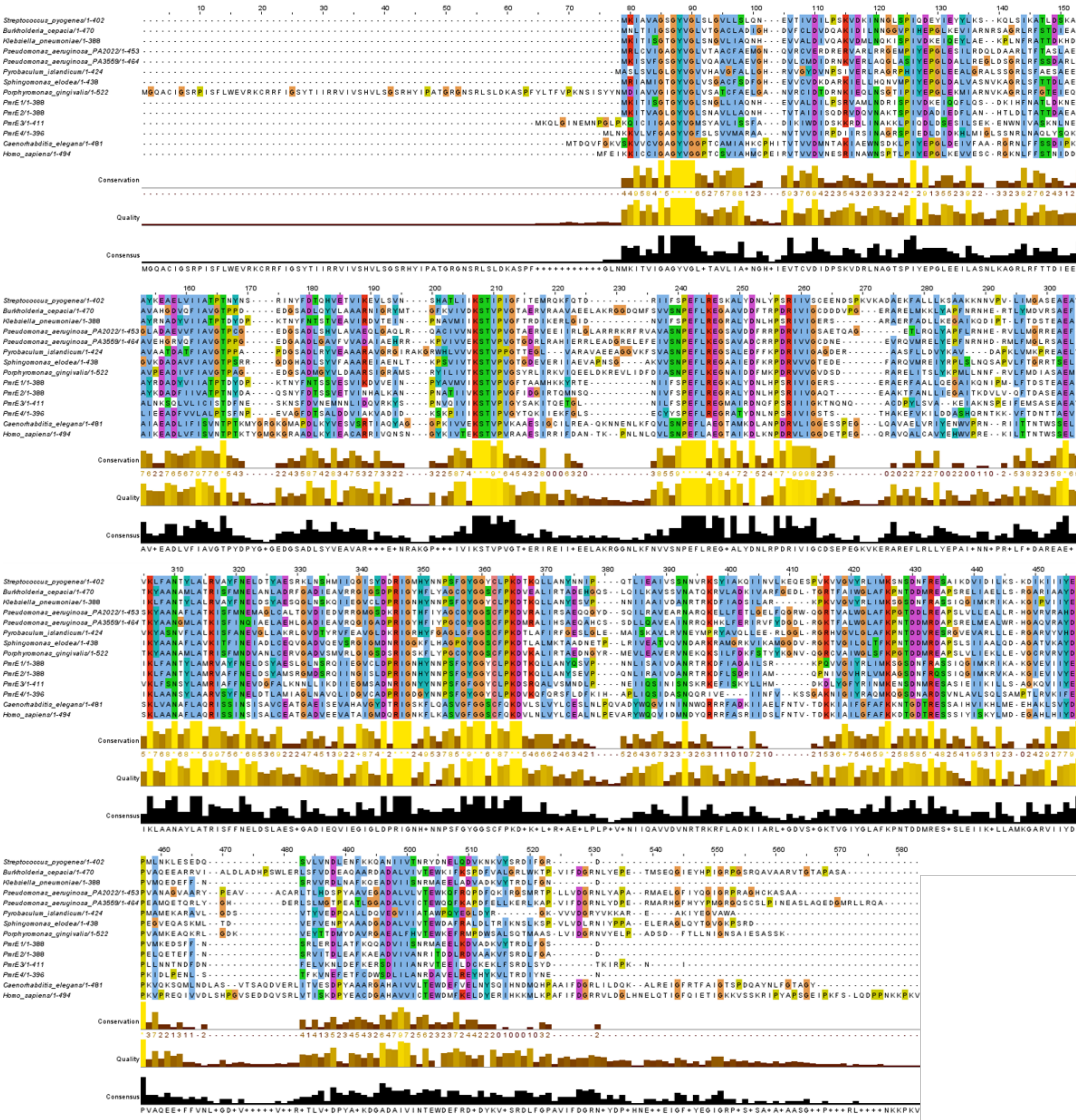

Figure S3. The sequence alignments of PmrE proteins. Previously characterized UGDH proteins were aligned with PmrE1–4 proteins.

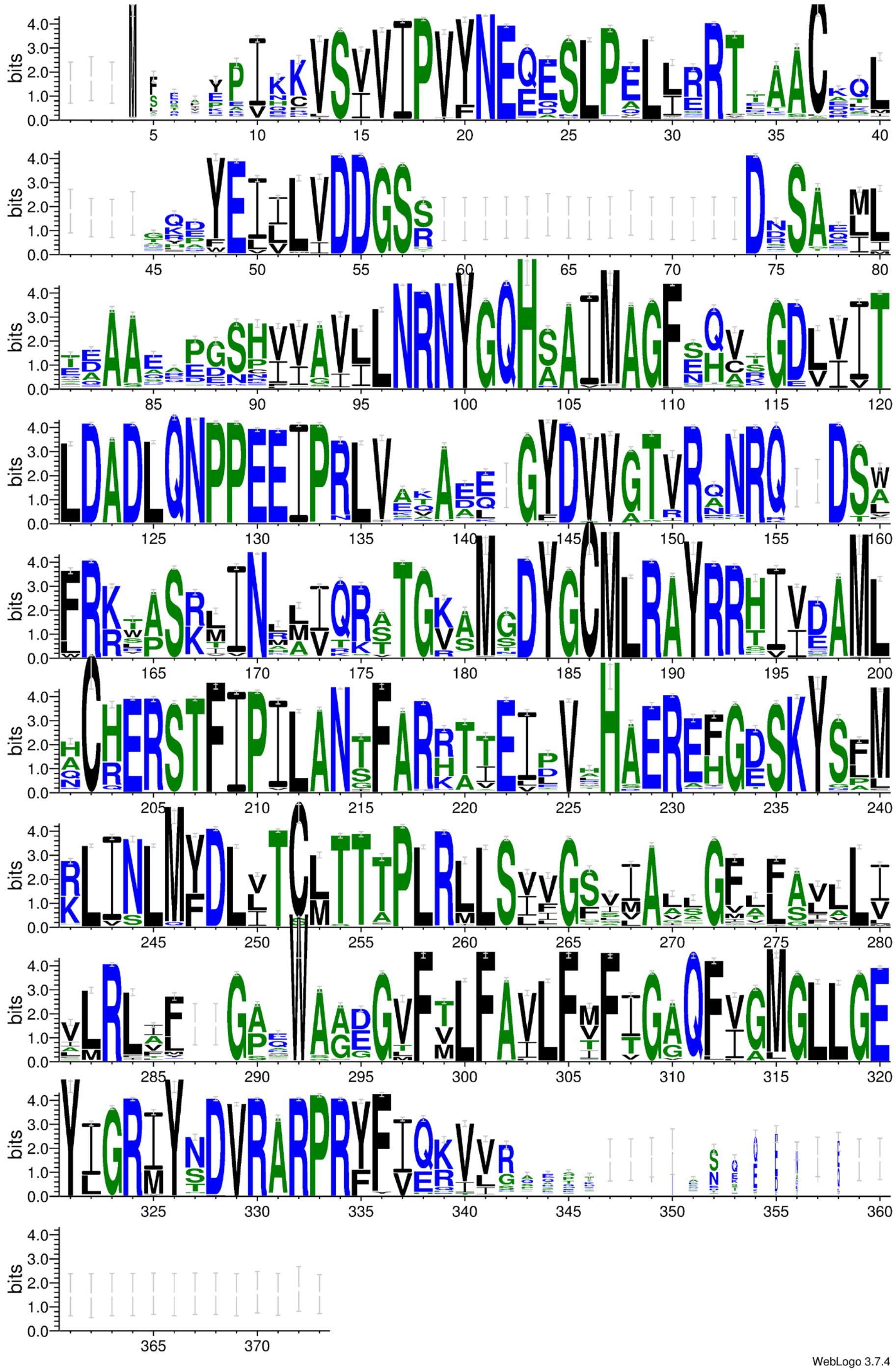

Figure S4. WebLogo frequency plot of amino acids at PmrF1-like proteins.

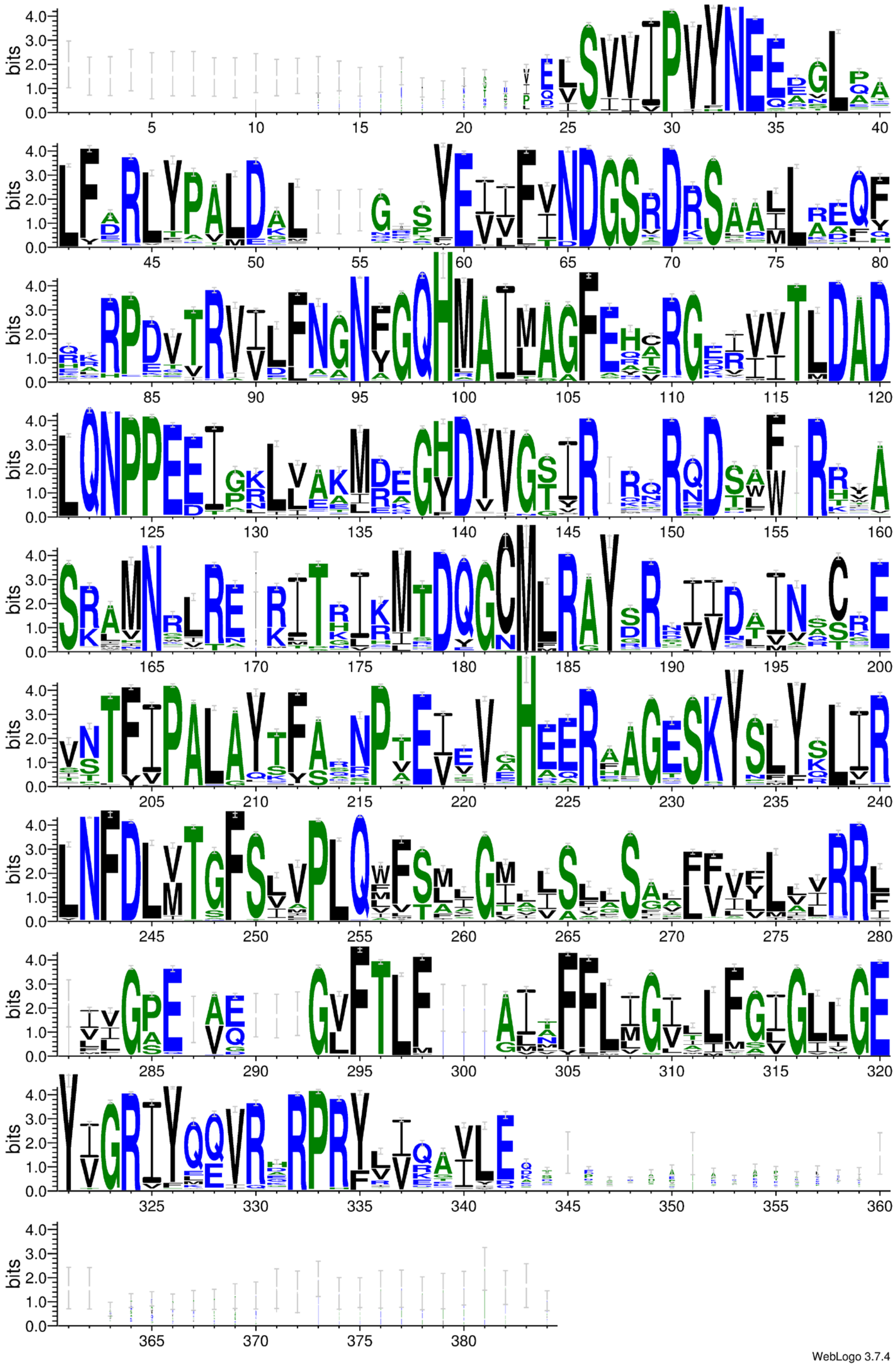

Figure S5. WebLogo frequency plot of amino acids at PmrF2-like proteins.

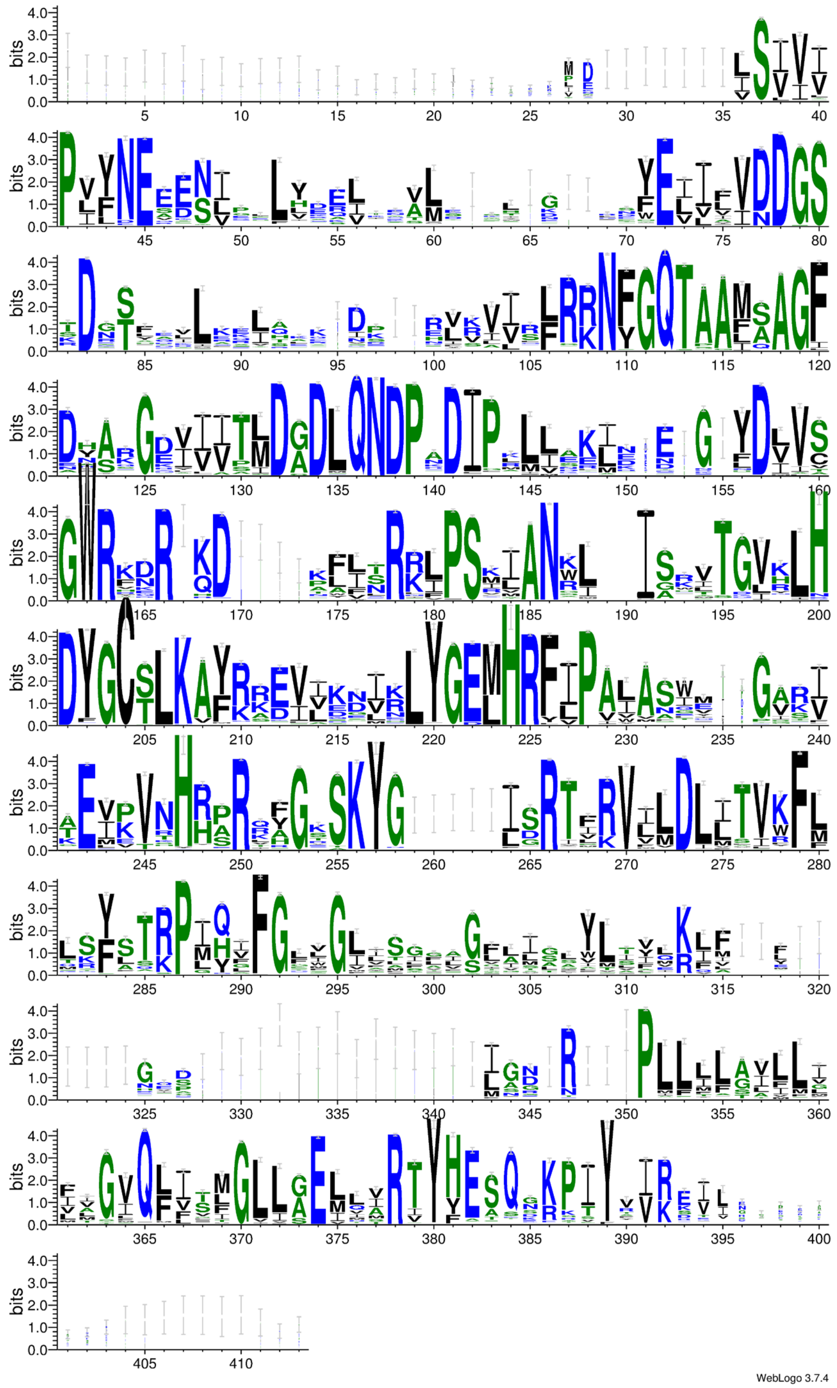

Figure S6. WebLogo frequency plot of amino acids at PmrF3-like proteins.

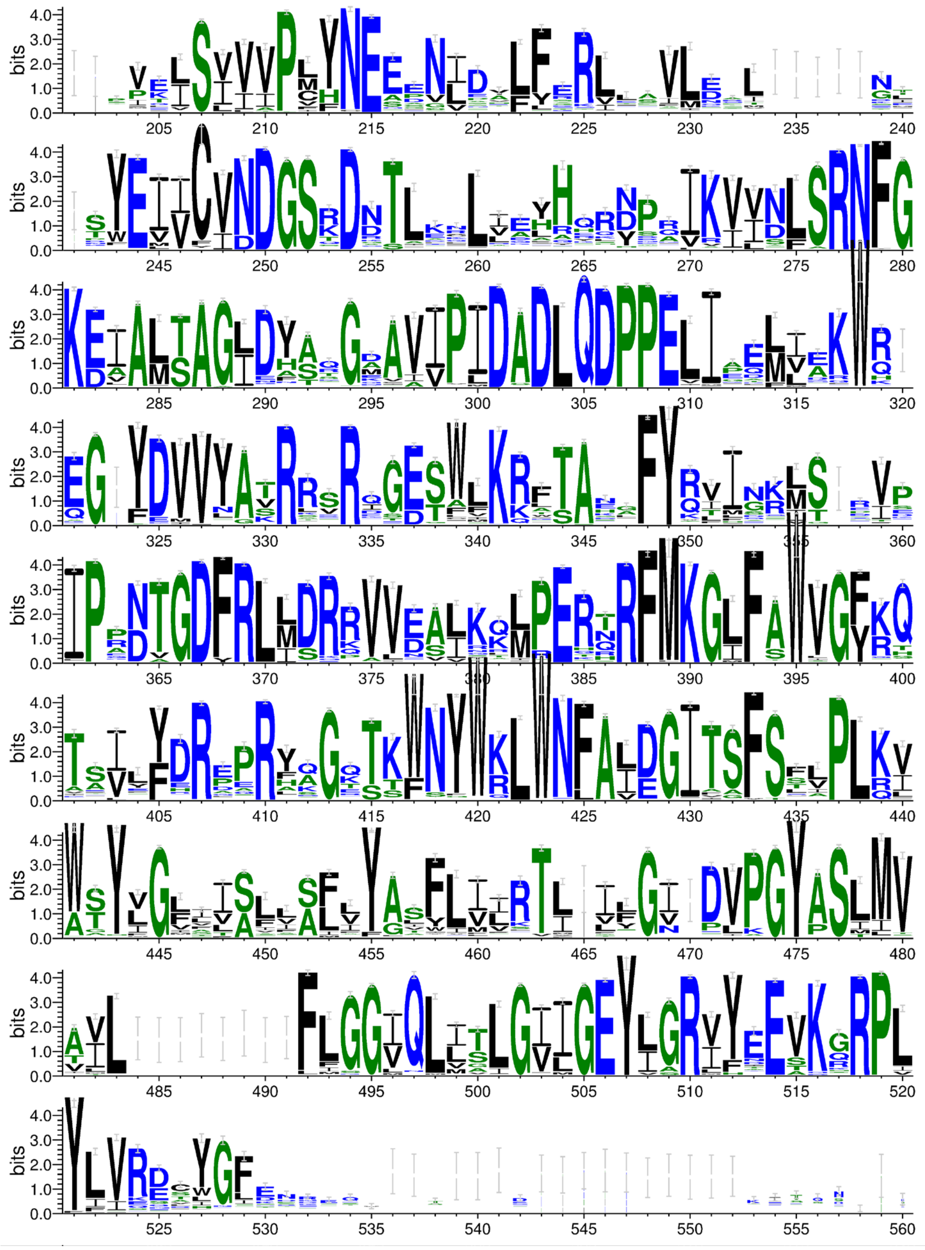

Figure S7. WebLogo frequency plot of amino acids at GtrB-like proteins.

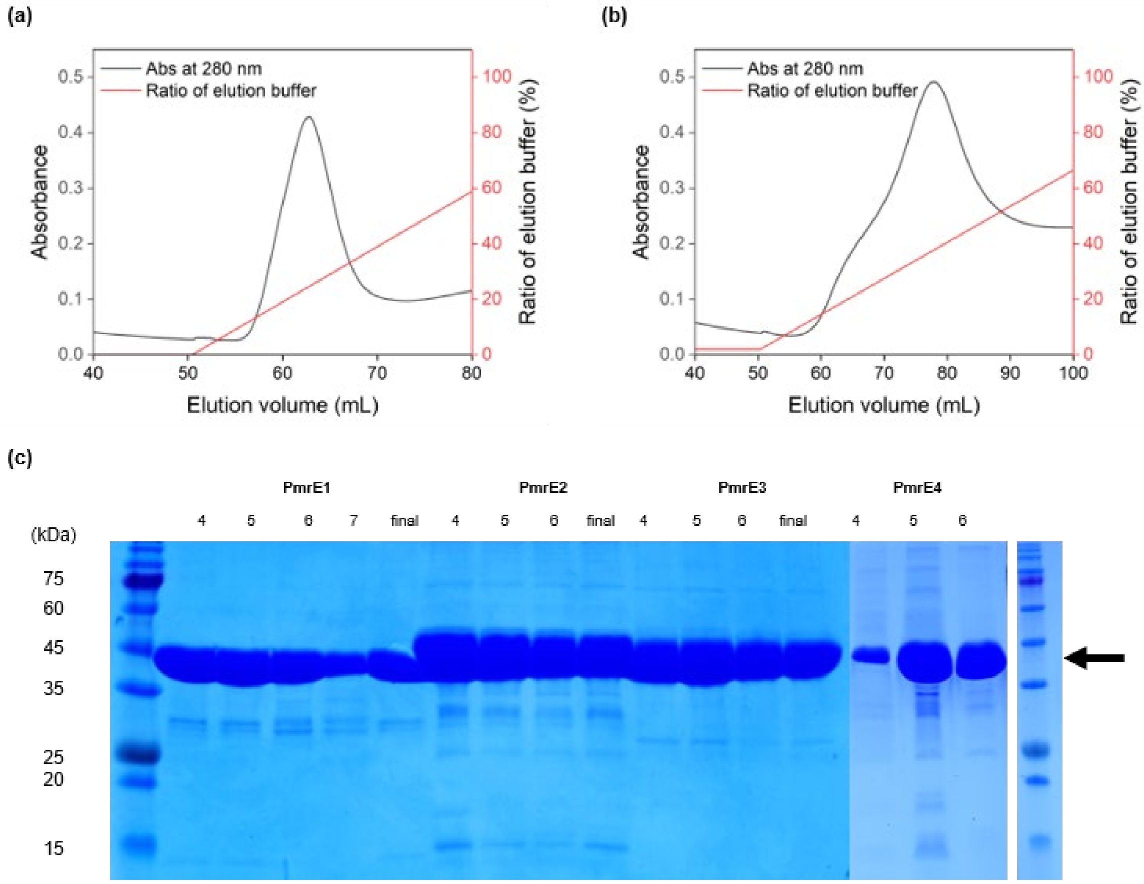

Figure S8. The purification of PmrE proteins. The representative FPLC traces of (a) PmrE1 and (b) PmrE2 in Ni-affinity chromatography and (c) SDS-PAGE analysis of the purified PmrE1–4 proteins. The arrow indicates the size of the desired proteins.

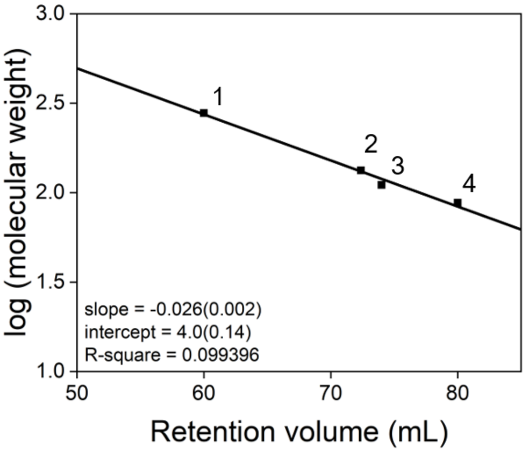

Figure S9. The calibration curve of size-exclusion chromatography to determine oligomeric state of PmrE and PmrF proteins. Proteins used for calibration are acetyltransferase from *Bacillus anthracis* (278.90 kDa), 2-keto-3-deoxyluconate aldolase from *Sulfolobus solfataricus* (133.36 kDa), DHRS6 from *Homo sapiens* (110.28 kDa), and phosphoheptose isomerase from *Pseudomonas aeruginosa* (87.68 kDa).

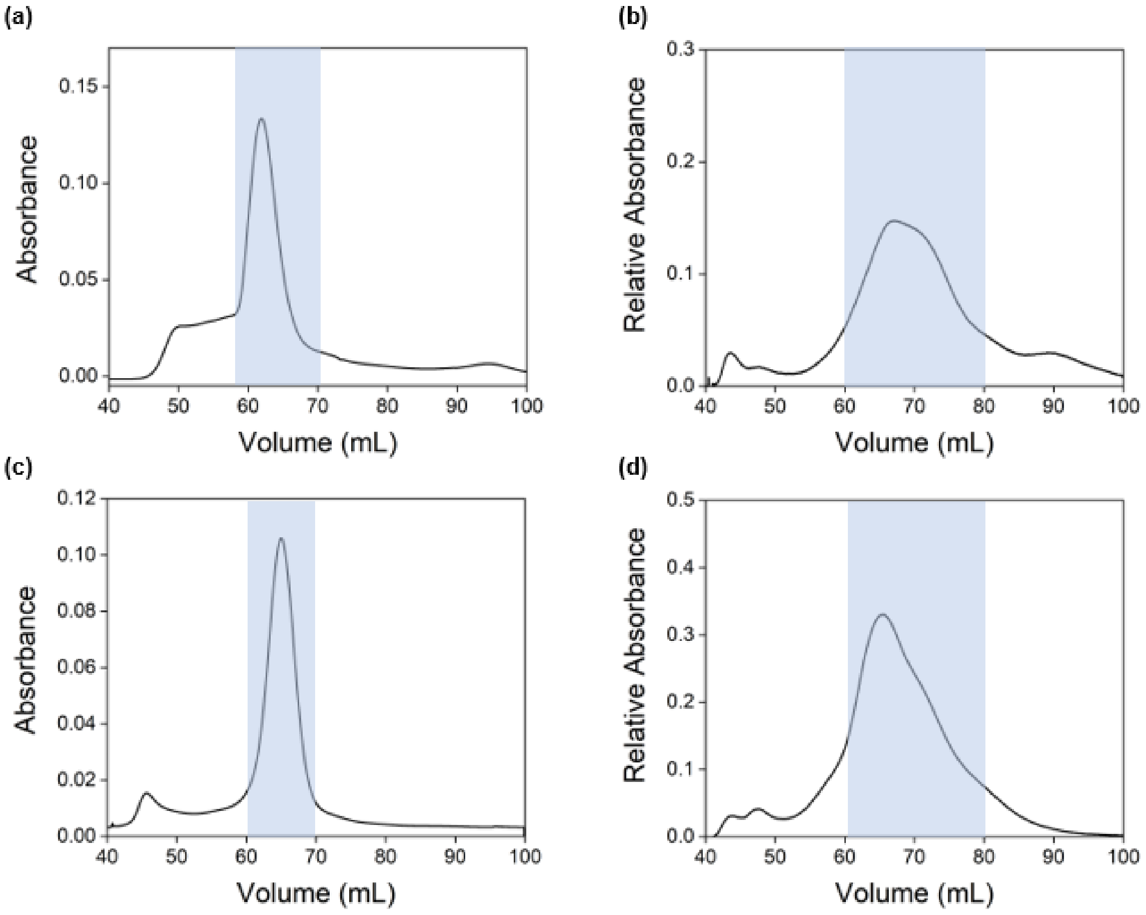

Figure S10. The size-exclusion chromatography of PmrE proteins. (a) PmrE1 (b) PmrE2 (c) PmrE3 (d) PmrE4. The highlighted fractions were used for the activity assays.

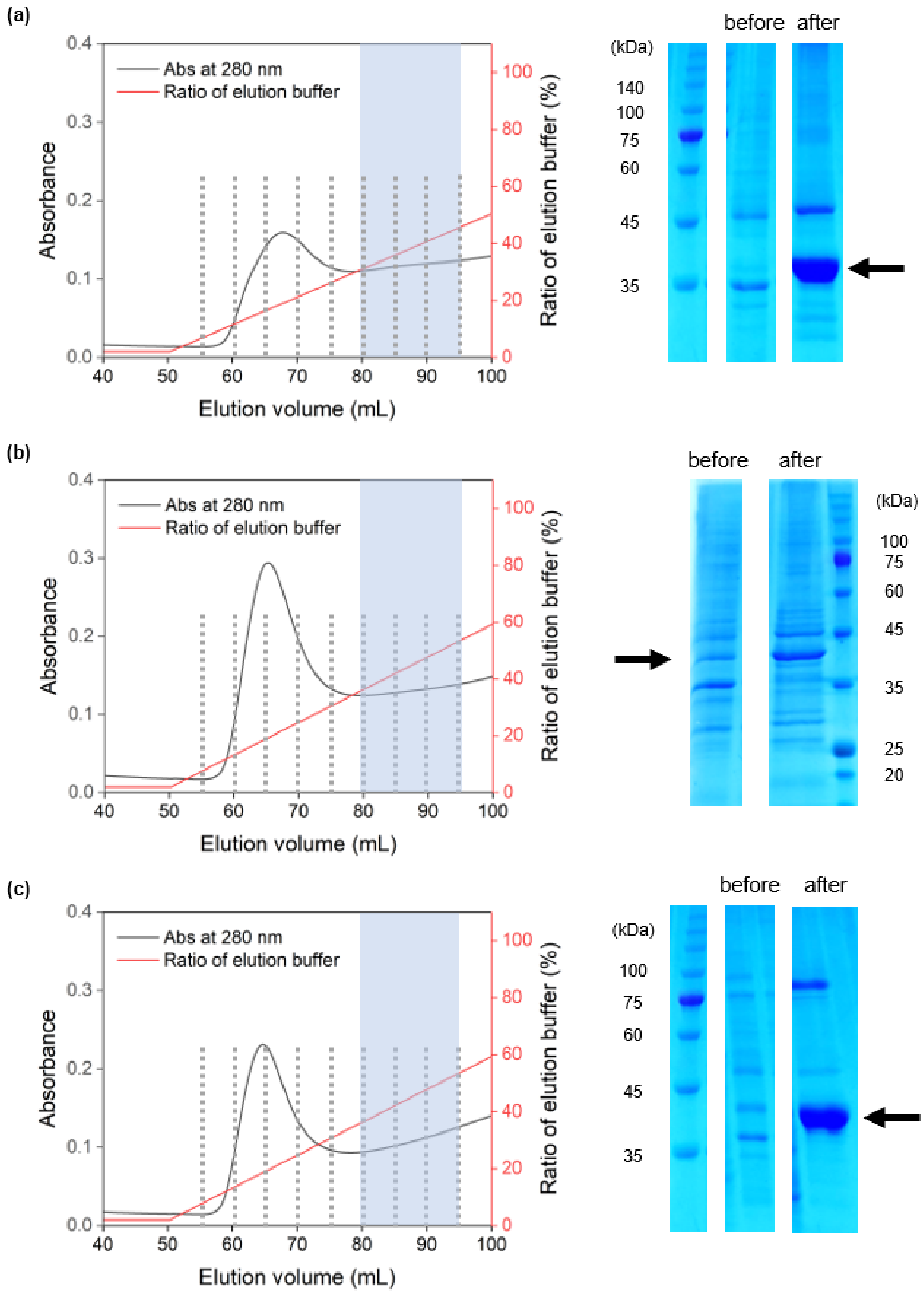

Figure S11. Purification of PmrF1–3 proteins. (a) PmrF1 (b) PmrF2 (c) PmrF3. (left) Representative FPLC traces in His-tag affinity chromatography (right) SDS-PAGE analysis before and after purification shown in (a). The arrows indicate the size of the desired proteins.

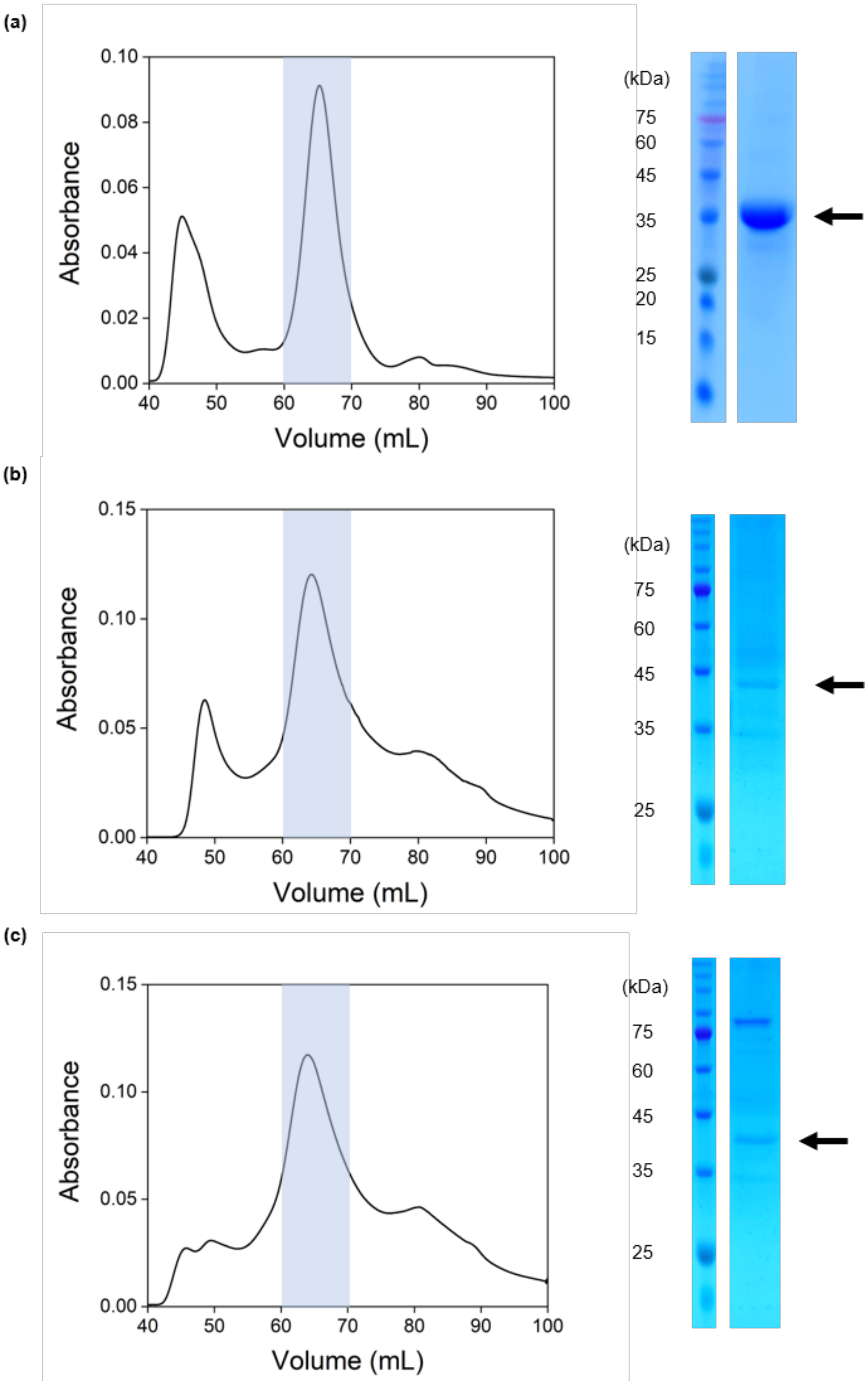

Figure S12. The size-exclusion chromatography of PmrF1–3 proteins. Representative FPLC traces and SDS-PAGE analysis of (a) PmrF1 (b) PmrF2 (c) PmrF3. In (c), a significant fraction of the protein was aggregated after size-exclusion chromatography, and the remaining soluble fraction was applied for SDS-PAGE. The arrows indicate the size of the desired proteins.

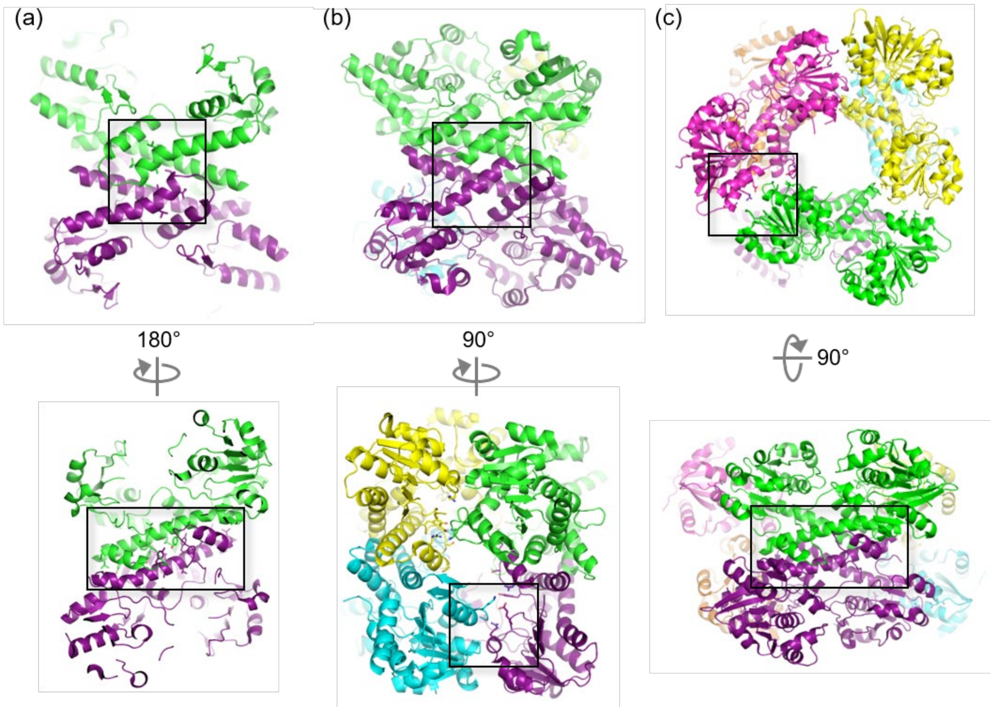

Figure S13. The structural analysis of PmrE-like proteins. Representative X-ray crystal structures of UGDH shown as (a) dimer (PDB 3PHL) (b) tetramer (PDB 3GG2) (c) hexamer (PDB 4RJT). The PPI domains are highlighted with black boxes.

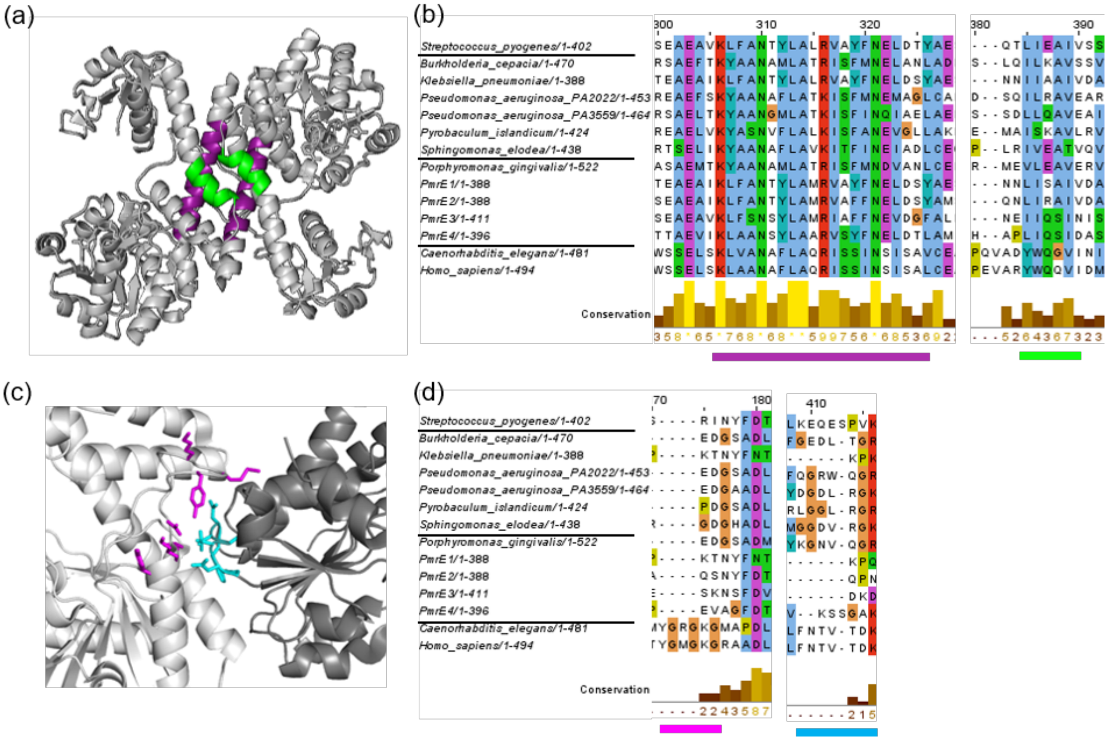

Figure S14. The structure and sequence analysis of PmrE-like proteins. (a) Two PPI domain for dimerization in *Klebsiella pneumoniae* UGDH (PDB 3PHL) colored in purple and green. (b) Multiple sequence alignment of UGDH, representing two PPI domains for dimerization, K(L|Y)(A|F)ANx(Y|F)LAx(R|K)(I|V)(S|A)(F|Y|S)(F|I)N(E|S)(L|I|V)xx(L|Y) and (I|L)(I|L)xA (I|V) (c) The PPI domain for hexamerization shown in human UGDH (PDB 4RJT). The key residues are shown with magenta or cyan sticks. (d) Multiple sequence alignment of PPI domain of UGDHs that form hexamer.

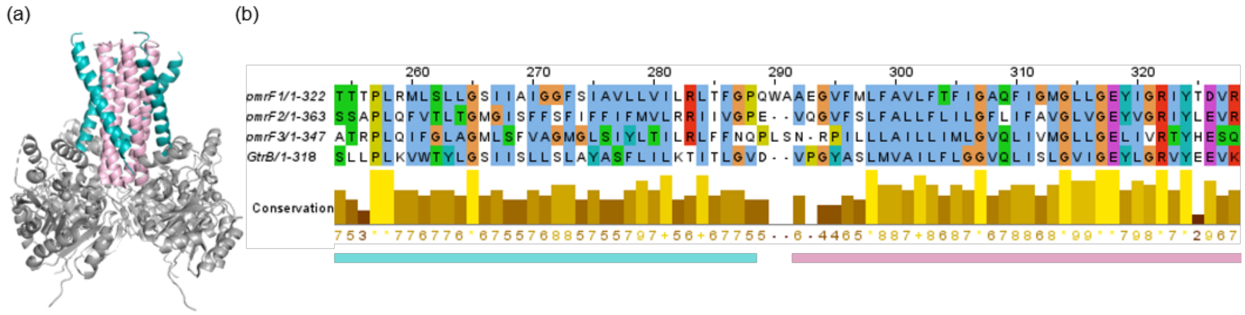

Figure S15. Structure and sequence analysis of *pmrF* genes. (a) The crystal structure of GtrB (PDB 5EKE). The transmembrane PPI domains are colored in cyan and light magenta. (b) Multiple sequence alignment of *pmrF*1–3 with GtrB, showing the PPI domains.

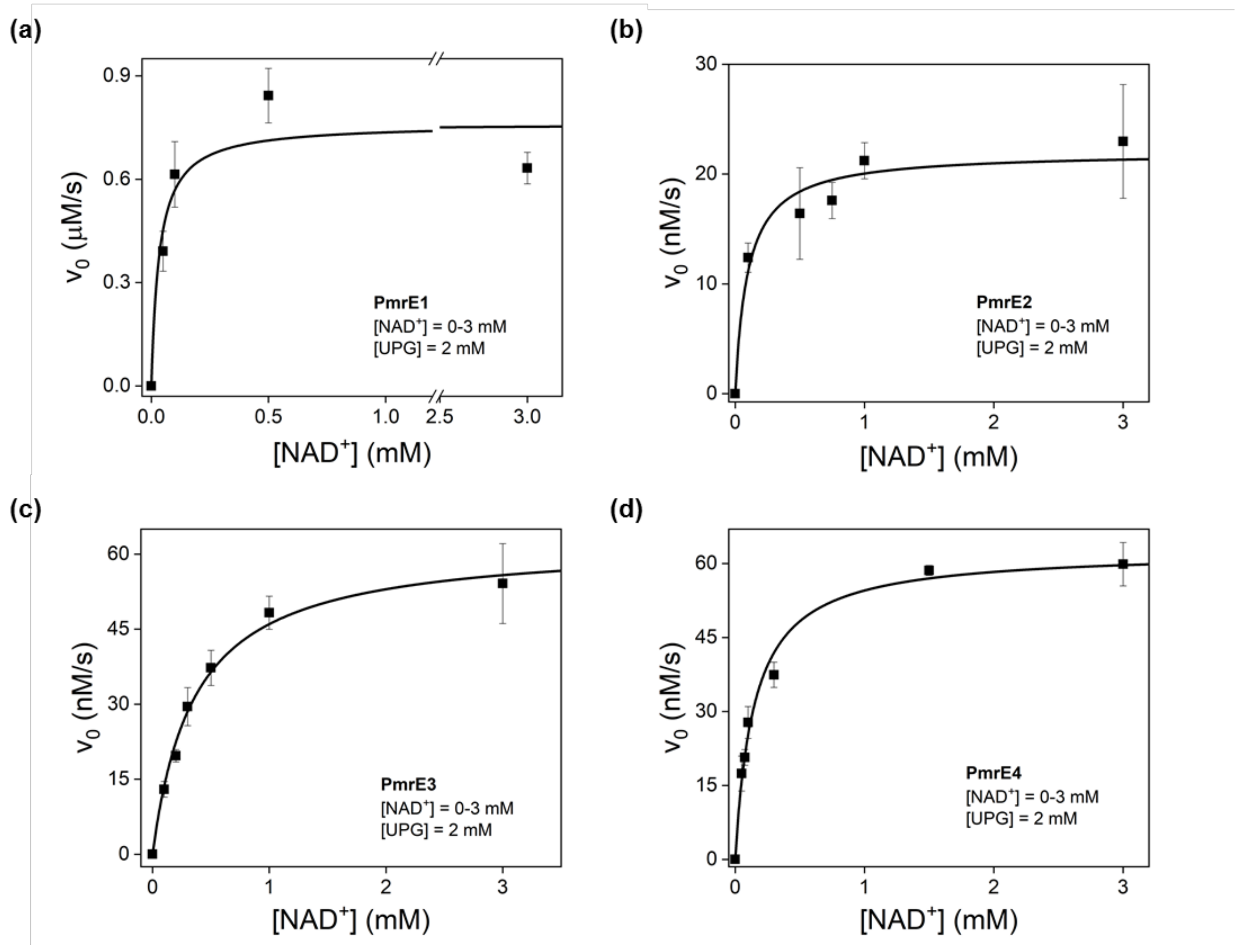

Figure S16. The steady-state kinetic analysis of PmrE1–4 with various concentrations of NAD^+. (a) PmrE1 (b) PmrE2 (c) PmrE3 (d) PmrE4.

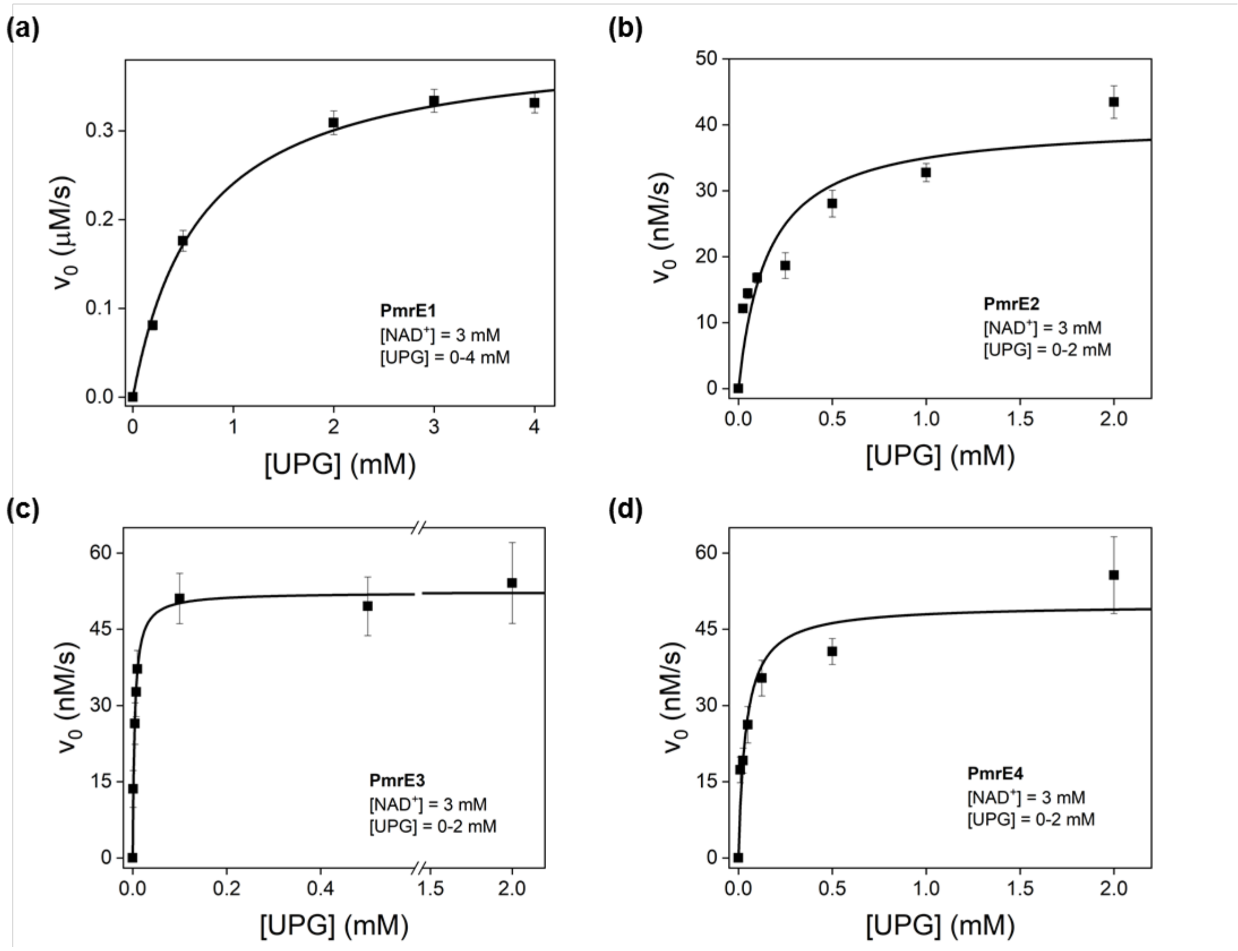

Figure S17. The steady-state kinetic analysis of PmrE1–4 with various concentrations of UPG. (a) PmrE1 (b) PmrE2 (c) PmrE3 (d) PmrE4.

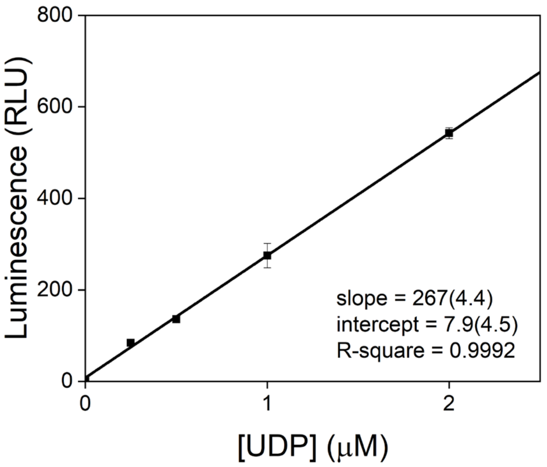

Figure S18. Standard curve of luminescence intensity versus UDP concentration.

References

1. Campbell RE, Sala RF, van de Rijn I, Tanner ME. 1997. Properties and Kinetic Analysis of UDP-glucose Dehydrogenase from Group A Streptococci: IRREVERSIBLE INHIBITION BY UDP-CHLOROACETOL*. J Biol Chem 272:3416-3422.
2. Rocha J, Popescu AO, Borges P, Mil-Homens D, Moreira LM, Sá-Correia I, Fialho AM, Frazão C. 2011. Structure of Burkholderia cepacia UDP-glucose dehydrogenase (UGD) BceC and role of Tyr10 in final hydrolysis of UGD thioester intermediate. J Bacteriol 193:3978-87.
3. Sieberth V, Rigg GP, Roberts IS, Jann K. 1995. Expression and characterization of UDPGlc dehydrogenase (KfiD), which is encoded in the type-specific region 2 of the Escherichia coli K5 capsule genes. J Bacteriol 177:4562-5.
4. Chen YY, Ko TP, Lin CH, Chen WH, Wang AH. 2011. Conformational change upon product binding to Klebsiella pneumoniae UDP-glucose dehydrogenase: a possible inhibition mechanism for the key enzyme in polymyxin resistance. J Struct Biol 175:300-10.
5. Hung RJ, Chien HS, Lin RZ, Lin CT, Vatsyayan J, Peng HL, Chang HY. 2007. Comparative analysis of two UDP-glucose dehydrogenases in Pseudomonas aeruginosa PAO1. J Biol Chem 282:17738-48.
6. Sakuraba H, Kawai T, Yoneda K, Ohshima T. 2012. Structure of a UDP-glucose dehydrogenase from the hyperthermophilic archaeon Pyrobaculum islandicum. Acta Crystallogr F68:1003-1007.
7. Barbas A, Popescu A, Frazão C, Arraiano CM, Fialho AM. 2013. Rossmann-fold motifs can confer multiple functions to metabolic enzymes: RNA binding and ribonuclease activity of a UDP-glucose dehydrogenase. Biochem Biophys Res Commun 430:218-24.
8. Riegert AS, Raushel FM. 2021. Functional and Structural Characterization of the UDP-Glucose Dehydrogenase Involved in Capsular Polysaccharide Biosynthesis from Campylobacter jejuni. Biochemistry 60:725-734.
9. Egger S, Chaikuad A, Kavanagh KL, Oppermann U, Nidetzky B. 2011. Structure and mechanism of human UDP-glucose 6-dehydrogenase. J Biol Chem 286:23877-87.
10. Kadirvelraj R, Custer GS, Keul ND, Sennett NC, Sidlo AM, Walsh RM, Wood ZA. 2014. Hysteresis in Human UDP-Glucose Dehydrogenase Is Due to a Restrained Hexameric Structure That Favors Feedback Inhibition. Biochemistry 53:8043-8051.
11. Kandiba L, Eichler J. 2016. AglM and VNG1048G, Two Haloarchaeal UDP-Glucose Dehydrogenases, Show Different Salt-Related Behaviors. Life (Basel) 6.
12. Ge X, Penney LC, van de Rijn I, Tanner ME. 2004. Active site residues and mechanism of UDP-glucose dehydrogenase. Eur J Biochem 271:14-22.
13. Chen YY, Ko TP, Chen WH, Lo LP, Lin CH, Wang AH. 2010. Conformational changes associated with cofactor/substrate binding of 6-phosphogluconate dehydrogenase from Escherichia coli and Klebsiella pneumoniae: Implications for enzyme mechanism. J Struct Biol 169:25-35.
14. Granja AT, Popescu A, Marques AR, Sá-Correia I, Fialho AM. 2007. Biochemical characterization and phylogenetic analysis of UDP-glucose dehydrogenase from the gellan gum producer Sphingomonas elodea ATCC 31461. Appl Microbiol Biotechnol 76:1319-27.

15. Wu MM, Huang HD, Li GQ, Zhou JF, Ma T. 2015. Biochemical characterization and functional analysis of UDP-glucose dehydrogenase, in the synthesis of biopolymer Ss from Sphingomonas sanxanigenens NX02. Prikl Biokhim Mikrobiol 51:30-6.
16. Li J, Rayner CR, Nation RL, Owen RJ, Spelman D, Tan KE, Liolios L. 2006. Heteroresistance to colistin in multidrug-resistant Acinetobacter baumannii. Antimicrob Agents Chemother 50:2946-50.
17. Lu X, Hu Y, Luo M, Zhou H, Wang X, Du Y, Li Z, Xu J, Zhu B, Xu X, Kan B. 2017. MCR-1.6, a New MCR Variant Carried by an IncP Plasmid in a Colistin-Resistant Salmonella enterica Serovar Typhimurium Isolate from a Healthy Individual. Antimicrob Agents Chemother 61.
18. Liu BT, Song FJ, Zou M, Hao ZH, Shan H. 2017. Emergence of Colistin Resistance Gene mcr-1 in Cronobacter sakazakii Producing NDM-9 and in Escherichia coli from the Same Animal. Antimicrob Agents Chemother 61.
19. Landman D, Bratu S, Alam M, Quale J. 2005. Citywide emergence of Pseudomonas aeruginosa strains with reduced susceptibility to polymyxin B. J Antimicrob Chemother 55:954-957.
20. Zheng B, Dong H, Xu H, Lv J, Zhang J, Jiang X, Du Y, Xiao Y, Li L. 2016. Coexistence of MCR-1 and NDM-1 in Clinical Escherichia coli Isolates. Clin Infect Dis 63:1393-1395.
21. Chew KL, La MV, Lin RTP, Teo JWP. 2017. Colistin and Polymyxin B Susceptibility Testing for Carbapenem-Resistant and mcr-Positive Enterobacteriaceae: Comparison of Sensititre, MicroScan, Vitek 2, and Etest with Broth Microdilution. J Clin Microbiol 55:2609-2616.
22. Zeng KJ, Doi Y, Patil S, Huang X, Tian GB. 2016. Emergence of the Plasmid-Mediated mcr-1 Gene in Colistin-Resistant Enterobacter aerogenes and Enterobacter cloacae. Antimicrob Agents Chemother 60:3862-3.
23. Malott RJ, Steen-Kinnaird BR, Lee TD, Speert DP. 2012. Identification of hopanoid biosynthesis genes involved in polymyxin resistance in Burkholderia multivorans. Antimicrob Agents Chemother 56:464-71.
24. Lin QY, Tsai YL, Liu MC, Lin WC, Hsueh PR, Liaw SJ. 2014. Serratia marcescens arn, a PhoP-regulated locus necessary for polymyxin B resistance. Antimicrob Agents Chemother 58:5181-90.
